# A Systematic Review on Predictors of Working Memory Training Responsiveness in Healthy Older Adults: Methodological Challenges and Future Directions

**DOI:** 10.3389/fnagi.2020.575804

**Published:** 2020-10-14

**Authors:** Anja Ophey, Mandy Roheger, Ann-Kristin Folkerts, Nicole Skoetz, Elke Kalbe

**Affiliations:** ^1^Department of Medical Psychology | Neuropsychology & Gender Studies, Center for Neuropsychological Diagnostics and Intervention (CeNDI), Faculty of Medicine and University Hospital Cologne, University of Cologne, Cologne, Germany; ^2^Department I of Internal Medicine, Center for Integrated Oncology Aachen Bonn Cologne Duesseldorf, Faculty of Medicine and University Hospital of Cologne, University of Cologne, Cologne, Germany

**Keywords:** prognostic review, systematic review, healthy aging, working memory training, training responsiveness, individual differences

## Abstract

**Background:** Research on predictors of working memory training responsiveness, which could help tailor cognitive interventions individually, is a timely topic in healthy aging. However, the findings are highly heterogeneous, reporting partly conflicting results following a broad spectrum of methodological approaches to answer the question “who benefits most” from working memory training.

**Objective:** The present systematic review aimed to systematically investigate prognostic factors and models for working memory training responsiveness in healthy older adults.

**Method:** Four online databases were searched up to October 2019 (MEDLINE Ovid, Web of Science, CENTRAL, and PsycINFO). The inclusion criteria for full texts were publication in a peer-reviewed journal in English/German, inclusion of healthy older individuals aged ≥55 years without any neurological and/or psychiatric diseases including cognitive impairment, and the investigation of prognostic factors and/or models for training responsiveness after targeted working memory training in terms of direct training effects, near-transfer effects to verbal and visuospatial working memory as well as far-transfer effects to other cognitive domains and behavioral variables. The study design was not limited to randomized controlled trials.

**Results:** A total of 16 studies including *n* = 675 healthy older individuals with a mean age of 63.0–86.8 years were included in this review. Within these studies, five prognostic model approaches and 18 factor finding approaches were reported. Risk of bias was assessed using the Quality in Prognosis Studies checklist, indicating that important information, especially regarding the domains study attrition, study confounding, and statistical analysis and reporting, was lacking throughout many of the investigated studies. Age, education, intelligence, and baseline performance in working memory or other cognitive domains were frequently investigated predictors across studies.

**Conclusions:** Given the methodological shortcomings of the included studies, no clear conclusions can be drawn, and emerging patterns of prognostic effects will have to survive sound methodological replication in future attempts to promote precision medicine approaches in the context of working memory training. Methodological considerations are discussed, and our findings are embedded to the cognitive aging literature, considering, for example, the cognitive reserve framework and the compensation vs. magnification account. The need for personalized cognitive prevention and intervention methods to counteract cognitive decline in the aging population is high and the potential enormous.

**Registration:** PROSPERO, ID CRD42019142750.

## Introduction

The promotion of healthy aging constitutes a major goal given the demographic change that the world's population is facing (Parish et al., 2019). One key aspect of healthy aging is the maintenance of cognitive functions by preventing or delaying the onset of clinically relevant cognitive dysfunction or even reversing age-related cognitive decline (Lustig et al., 2009). Cognitive decline is one of the most feared aspects in aging (Deary et al., 2009), as it reduces the quality of life of both the aging individual and his/her relatives and increases the burden on care providers and the public healthcare system. Decline of executive functions, working memory, processing speed, and memory—cognitive functions that are essential for everyday functioning—is the most prominent cognitive alteration in healthy aging (Paraskevoudi et al., 2018). Especially working memory, a capacity-limited system for short-term storage and manipulation of information, is of fundamental importance for general cognitive functioning and is seen as a key function and processing resource for other cognitive abilities (Salthouse, 1990; Chai et al., 2018).

Cognitive training interventions, as a non-pharmacological intervention and prevention method, have gained increased scientific interest (Lustig et al., 2009). A recent meta-analysis of Chiu et al. (2017) on broad cognitive interventions in healthy older adults clearly indicated the potential of cognitive interventions to counteract cognitive decline. However, some issues such as the degree of transfer to untrained tasks and long-term effects remain a matter of debate. In this context, working memory has become a main target for cognitive training interventions. The role of working memory as a processing resource for other cognitive abilities (Salthouse, 1990; Chai et al., 2018) implies that working memory improvements after targeted working memory training (WMT) might naturally lead to positive transfer effects to other cognitive functions and even fluid intelligence (Au et al., 2015). Despite a general consensus regarding the effectiveness of targeted WMT regarding direct training effects (i.e., effects in trained working memory tasks over the course of training) and near-transfer effects (i.e., effects in untrained working memory tasks), evidence on far-transfer effects (i.e., effects in untrained domains) for different populations including healthy older adults has not convincingly been shown (for recent meta-analyses see e.g., Melby-Lervåg et al., 2016; Weicker et al., 2016; Soveri et al., 2017; Sala et al., 2019; Teixeira-Santos et al., 2019). Given those heterogeneous results concerning effects after WMT, identifying modifying, so-called prognostic or moderating, factors (including both individual- and training-related characteristics) of WMT responsiveness seems highly relevant.

In general, a prognostic factor is defined as any measure that, among people with a given condition (e.g., the process of aging), is associated with a subsequent outcome (e.g., changes in cognition after certain interventions) (Riley et al., 2013). In prognostic research, prognostic factor finding studies and prognostic model studies are distinguished: prognostic factor finding studies aim at establishing one or several variables as independent prognostic factors associated with an outcome. In contrast, prognostic model studies identify more than one prognostic factor, assign relative weight to each prognostic factor, and estimate the model's predictive performance through calibration and discrimination (Moons et al., 2009). Identifying prognostic factors for individual treatment response to WMT would take into account individual differences in cognitive plasticity and following responsiveness to cognitive training interventions (Baltes and Lindenberger, 1988; Noack et al., 2009; Bürki et al., 2014). It would further contribute to the development of an encompassing approach in terms of a “personalized” or “precision medicine” (Hingorani et al., 2013) in healthy aging and the prevention of cognitive decline, for example, in the context of Alzheimer's disease (Reitz, 2016; Berkowitz et al., 2018).

The latest meta-analysis on WMT for healthy older adults (Teixeira-Santos et al., 2019) included a broad moderator analysis for WMT responsiveness. Despite training-related variables (e.g., training dose and length, number of sessions, training type), study population characteristics (e.g., age, education, general cognitive ability, baseline performance) were considered as moderating variables (Teixeira-Santos et al., 2019). The meta-analysis mainly identified training-related characteristics as moderating variables for WMT response: for example, longer training durations in hours were associated with smaller effect sizes across studies (Teixeira-Santos et al., 2019). Note, however, that whereas prognostic factors are, per definition, measured and investigated on an individual-person level, the moderator analysis approach within the standard meta-analytical approach investigates modifying factors on an aggregated, study-wide level, i.e., across many individuals (e.g., mean age of participants, mean years of education). Therefore, interindividual variance of those parameters and corresponding differential training outcomes within the single-study populations are neglected in the meta-analysis of Teixeira-Santos et al. (2019). A focus on research using prognostic approaches on a single-study level would therefore substantially expand upon already existing data.

Prognostic research on treatment responsiveness after WMT has received increasing interest on a single-study level as well. However, data are inconclusive yet, as findings are highly heterogeneous and inconsistent, and prognostic approaches are often considered as an add-on analysis beyond standard effectiveness evaluations only. It seems that, especially if an intervention did not yield an overall positive effect, researchers tend to exploratively analyze prognostic factors of training responsiveness. One could argue that conducting prognostic analyses on null effects might be dealing with pure noise. However, prognostic research is obliged to detangle predictors of systematic retest effects, such as practice effects or regression to the mean, from predictors of treatment response (Hingorani et al., 2013). Therefore, it is tremendously important to compare the prognostic factors between a control group and the group receiving the treatment of interest (Hingorani et al., 2013). To anticipate one weakness of prognostic research in the context of cognitive interventions including WMT so far, prognostic effects are often investigated with data of the experimental group only.

Two of the most frequently investigated prognostic factors for WMT responsiveness are baseline performance in working memory or the respective cognitive outcome and general cognitive ability (e.g., Zinke et al., 2014; Borella et al., 2017b; Matysiak et al., 2019). For both, inconsistent findings exist, which can be discussed within the compensation vs. magnification framework (Lövdén et al., 2012). Following the compensation account, individuals with lower baseline performance would show higher training benefits because they have more room for improvement. On the contrary, the magnification hypothesis constitutes that individuals with higher abilities would benefit most, as they have more resources “to acquire, implement, and sharpen effortful cognitive strategies” (Lövdén et al., 2012). Similar inconsistent evidence exists, e.g., for age (e.g., Borella et al., 2013, 2014, 2017b; Zinke et al., 2014; Simon et al., 2018) and other demographic factors such as education (Clark et al., 2016; Mondini et al., 2016; Borella et al., 2017b; Matysiak et al., 2019) and sex (Rahe et al., 2015; Matysiak et al., 2019; Roheger et al., 2019). Furthermore, motivational processes (West et al., 2008; Kalbe et al., 2018) and personality traits (Studer-Luethi et al., 2012; Double and Birney, 2016) might constitute important individual characteristics predicting training responsiveness as well. Finally, genetic variation (Brehmer et al., 2009; Bellander et al., 2011; Bäckman and Nyberg, 2013) and brain imaging parameters (Stern, 2009; Heinzel et al., 2014a) might reflect meaningful proxies for the potential to engage in cognitive plasticity following cognitive training interventions. To summarize, a broad spectrum of potential prognostic factors to predict individual training responsiveness is discussed; however, data are inconclusive yet. Therefore, systematic reviews and meta-analyses to summarize existing evidence about prognostic factors and models of individual treatment response in the context of cognitive interventions in general and WMT in particular are urgently needed but missing so far.

On the basis of the aforementioned considerations, the present systematic review aimed to systematically investigate prognostic factors and models for WMT responsiveness in healthy older adults. We further aimed to meta-analyze groups of “similar” prognostic effect measures to quantitatively investigate the predictive performance of the different prognostic factors. However, to anticipate one limitation of this work, data on prognostic factors after WMT were too heterogeneous and too poorly reported to conduct this meta-analysis after all.

Our systematic review question was defined using the PICOTS system as proposed by the Checklist for Critical Appraisal and Data Extraction for Systematic Reviews of Prediction Modeling Studies (CHARMS) (Moons et al., 2014; Debray et al., 2017; Riley et al., 2019). Our target population (P) consisted of healthy (i.e., absence of any neurological or psychiatric disease) older (aged ≥ 55 years) individuals. The target intervention (I) was single-domain WMT. No comparator factor (C) is being considered. The outcome variables (O) for this review are training and near-transfer effects to the domains of verbal and visuospatial working memory as well as far-transfer effects to other cognitive domains and behavioral variables, if applicable, operationalized with objective and standardized instruments, after targeted WMT. The timing (T) of recording the relevant variables is the baseline assessment for prognostic factors and all time points of measurement for outcome variables, including follow-ups. The setting (S) was supposed to be a non-clinical one to gain prognostic information on possibilities of enhancing cognitive functioning and the prevention of cognitive decline in cognitively healthy individuals.

## Methods

The preregistered review protocol of the present systematic review can be accessed through https://www.crd.york.ac.uk/PROSPERO/ (ID: CRD42019142750). The reporting follows the Preferred Reporting Items for Systematic Reviews and Meta-Analyses (PRISMA) guideline for systematic reviews and meta-analyses (Moher et al., 2009). The PRISMA checklists for abstracts and systematic reviews are displayed in Supplementary Material 1.

### Search Strategy

As prognostic studies are often not indexed, a broad and rather unspecific search filter was used (Riley et al., 2019). We conducted a systematic search throughout four online databases up to October 2019: MEDLINE Ovid, Web of Science Core Collection, CENTRAL, and PsycINFO. A series of keywords which were expected to appear in the title or the abstract of any study containing analyses on prognostic factors or models for WMT success was created. The keywords used can be grouped into three main categories. The first category aimed to identify studies including healthy older adults as participants (e.g., “healthy elderly,” “healthy aging,” “older adults”). The second category was used to detect a broad spectrum of interventional studies not only covering “working memory training” but also a broader spectrum of cognitive interventions (e.g., “cognitive training,” “reasoning training”) and even interventional studies *per se* (e.g., “training,” “intervention”). This broad intervention category was built to ensure the search strategy to cover all kinds of WMT that are differentially labeled in literature. The third category was included to ensure (working) memory to be a central construct of the included studies (“memory”). In addition to the systematic database search, the reference lists of all relevant full texts, review articles, and current treatment guidelines were hand-searched for further suitable articles. Further information and full search strings for each database can be obtained from Supplementary Material 2.

### Study Selection and Data Extraction

Title and abstract screening with predefined eligibility criteria was conducted by two reviewers (AKF and MR or AO and MR) in Covidence Systematic Review Software (Veritas Health Innovation, available at www.covidence.org). Then, the full-text articles were screened for final inclusion in the systematic review by two reviewers (AO and MR). If a full text was not available online, we contacted the corresponding authors and asked them to provide the full-text publication within 2 weeks of time. If no consensus was reached between the two reviewers (AO and MR), the plan was to discuss the case with a third author (NS) until a final consensus was reached; however, this option was not needed. Relevant data considering general study characteristics (e.g., participants' demographics, WMT features) and prognostic factor and/or model analyses were independently extracted by two reviewers (AO and MR) according to the CHARMS checklist (Moons et al., 2014).

### Eligibility Criteria

The inclusion criteria for our systematic review were (i) full-text research article publication until October 2019 in a peer-reviewed journal in English or German, (ii) inclusion of healthy older individuals aged ≥55 years without any neurological and/or psychiatric diseases including cognitive impairment (mild cognitive impairment or dementia) as well as uncorrected seeing or hearing impairments assessed *via* self-report, and (iii) investigation of prognostic factors and/or models for training responsiveness in terms of direct training and near-transfer effects to verbal and visuospatial working memory as well as far-transfer effects to other cognitive domains and behavioral variables, operationalized with objective and standardized instruments, after targeted WMT.

Age of ≥55 years was chosen as a cutoff, as we, on the one hand, wanted to provide an objective age cutoff for individuals within the included studies and, on the other hand, did not want to exclude studies including healthy older individuals just below the frequently used cutoff of ≥60 years (e.g., Soveri et al., 2017; Sala et al., 2019). Targeted WMT was defined as a cognitive training either computerized, with paper–pencil tasks, or mixed, which is administered either on personal devices or in individual or group settings, with a minimum of two training sessions. When multi-domain trainings were examined, working memory had to be the main component of the program (defined as being the main target in at least 80% of the exercises). Verbal and visuospatial working memory, i.e., direct training and near-transfer effects, were defined as primary outcomes, with direct training effects constituting effects in trained working memory tasks over the course of training and with near-transfer effects constituting effects in untrained working memory tasks. Other cognitive far-transfer outcomes (i.e., effects in untrained cognitive domains, e.g., global cognition, memory, fluid intelligence, executive functions, attention) and clinical and patient-centered outcomes (e.g., depressive symptoms, quality of life) were considered as secondary outcomes. Both primary and secondary outcomes needed to be assessed with established and objective psychometric instruments.

For the systematic review, we considered all prognostic factors (e.g., sociodemographic factors, cognitive abilities at the entry of training, brain imaging parameters, genetic parameters, personality traits, training-related characteristics), which investigate critical aspects of WMT responsiveness. As outlined in the introduction, a prognostic factor is defined as any measure that, among people with a given condition (e.g., the process of aging), is associated with a subsequent outcome (e.g., changes in cognition after certain interventions) (Riley et al., 2013). Prognostic factor finding studies aim at establishing one or several variables as independent prognostic factors associated with an outcome. In contrast, prognostic model studies identify more than one prognostic factor, assign relative weights to each prognostic factor, and estimate the model's predictive performance through calibration and discrimination (Moons et al., 2009). We included all studies investigating prognostic factors and/or prognostic models regardless of whether or not significant general training effects and/or significant relationships between prognostic factors and training responsiveness were found.

### Quality Assessment

Using the Quality in Prognosis Studies (QUIPS) checklist (Hayden et al., 2013), risk of bias of the included studies was examined independently by two reviewers (AO and MR) across six domains: study participation, study attrition, prognostic factor measurement, outcome measurement, adjustment for other prognostic factors, statistical analyses, and reporting. Each domain was overall rated with high, moderate, or low risk, depending on the rating in the corresponding items. A detailed description of the QUIPS checklist, including each item and the overall judgment rules implemented by the two reviewers, is presented in Supplementary Material 3. Instead of using two different risk of bias assessment tools [QUIPS (Hayden et al., 2013) for prognostic factor finding studies and Prediction Model Risk of Bias Assessment Tool (Wolff et al., 2019) for prognostic model studies], risk of bias of both prognostic factor finding and prognostic model studies was assessed with the QUIPS tool to get a comparable risk of bias rating.

### Data Analysis

Initially and as stated in the pre-registration of the study, we aimed to meta-analyze groups of “similar” prognostic effect measures with a random effects approach to investigate the predictive performance of the different prognostic factors. However, after data extraction, we had to ascertain that data on prognostic factors after WMT were too heterogeneous and too poorly reported to conduct this meta-analysis. The main reason was that we were not able to compute comparable effect size measures (e.g., odds ratios, hazard ratios) to meta-analyze the prognostic effects reported in the studies due to the fact that either data were not reported and could not be assessed within studies or data were not consistent enough across studies to pool the results. Therefore, the systematic review focused on the qualitative directionality of the prognostic effects reported in the included studies rather than their magnitude.

## Results

### Study Flow

**Figure 1:**
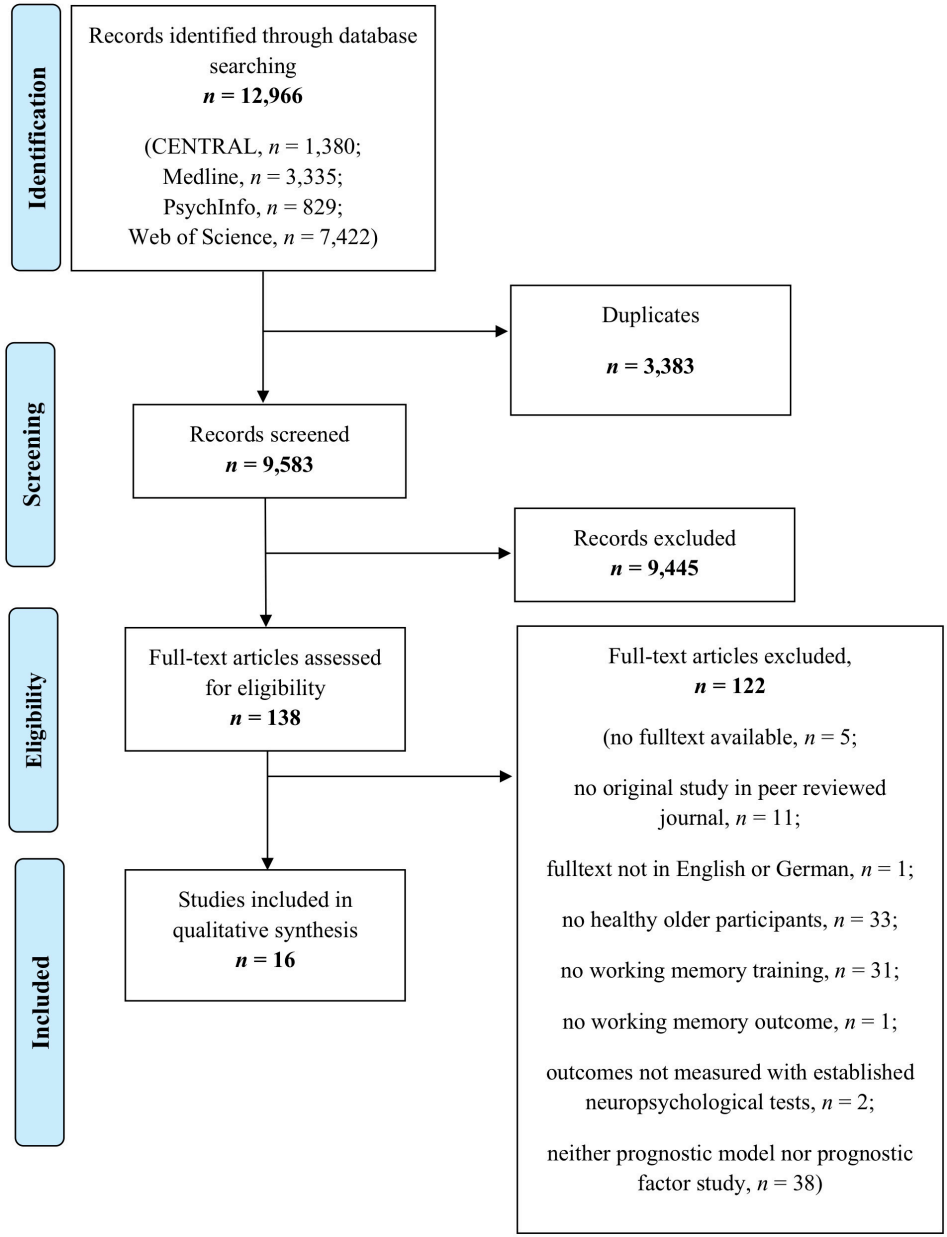
Preferred reporting items for systematic reviews and meta-analyses flow diagram.

A total of 12,966 records were identified through our database search. After removing duplicates, titles and abstracts of 9,583 records were screened for eligibility. As prognostic analyses are often not indexed, title and abstract screening focused on the content-related criteria “healthy older individuals” and “working memory training.” Thus, 138 full texts were screened for eligibility. Finally, *n* = 16 studies were included in the present systematic review [for details on study flow and reasons for exclusion, see Figure 1 (PRISMA flow diagram)].

### Descriptive Characteristics of the Included Studies

Within the 16 studies, *n* = 675 healthy older individuals, with age ranging from a mean of 63.0 years (Brehmer et al., 2011) to 86.8 years (Zinke et al., 2012), were investigated, of which 63% were women. Years of formal education ranged from a mean of 5.72 years (Borella et al., 2013) to 18.65 years (Tusch et al., 2016). Throughout the studies, different training regimes that varied in terms of setting, number of sessions, total time of training, and training tasks were applied. The number of training sessions ranged from three (Borella et al., 2013, 2014, 2017a,b,c; Brum et al., 2018) to 25 (Brehmer et al., 2011; McAvinue et al., 2013; Tusch et al., 2016; Simon et al., 2018; Matysiak et al., 2019), with the total time of training ranging from 105 min (Brum et al., 2018) to 1,000 min (Tusch et al., 2016); 44% of trainings addressed verbal working memory only and 50% followed a mixed approach, addressing both verbal and visuospatial working memory. Only one study conducted a multi-domain WMT, as next to working memory tasks one executive control task was included within the training regime (Zinke et al., 2014). All training regimes were conceptualized as adaptive, except for those studies in which adaptivity was investigated as a prognostic factor for WMT responsiveness (Brehmer et al., 2011; Tusch et al., 2016; Simon et al., 2018; Weicker et al., 2018).

In total, nine studies applied digital WMT: four studies used commercially available digital WMT programs (Cogmed and WOME/ RehaCom®) (Brehmer et al., 2011; Tusch et al., 2016; Simon et al., 2018; Weicker et al., 2018), three studies used a digital n-back training (Heinzel et al., 2014a,b; Matysiak et al., 2019), and two used a study–individual composition of digital WMT tasks (McAvinue et al., 2013; Borella et al., 2014). Five studies used a WMT with the Categorization Working Memory Span (CWMS) Task based on audio recordings (Borella et al., 2013, 2017a,b,c; Brum et al., 2018); however, all of these studies were conducted by the same group of researchers. Only two studies used paper–pencil WMT (Zinke et al., 2012, 2014) (for details on the study, participants, and training characteristics, see Table 1).

**Table 1:** Study objectives, participants' demographics, and working memory training characteristics.

**Study**	**Analysis**		**Prognostic factors**		**Participants**					**Training**	
**Author** ** (year)**	**Prognostic model**	**Factor finding**	**Individual-related**	**Training-related**	***n*^a^**	**Age** ** (in years)**	**Sex**	**Education** ** (in years)**	**Global cognition**	**Total time of training (in minutes) and setting**	**Description of training**
Borella et al. (2013)		X	X		38	Young-old^b^ 69.00 (3.18), 65-75; old-old79.22 (3.49), 75–87	Young-old 13 ♀, 7 ♂; old-old 12 ♀, 6 ♂	Young-old 9.40 (3.95); old-old 5.72 (2.52)	Vocabulary score WAIS-R^c^, max. 70; Young-old 46.65 (8.64); old-old 42.72 (9.04)	180 (three sessions of 60 min over 2 weeks) individual setting	Adaptive verbal working memory training with the Categorization Working Memory Span (CWMS^d^) Task *via* audio-recordings
Borella et al. (2014)		X	X	X	40 40	Young-old 69.90 (2.79), 65–75;old-old79.60 (2.28), 76–84	n.a.	Young-old 10.65 (2.50);old-old 8.75 (1.33)	Vocabulary score WAIS-R^c^, max. 70; young-old 49.25 (5.82); old-old 50.15 (4.57)	180 (three sessions of 60 min over 2 weeks)individual setting	Adaptive visuospatial working memory training with a computerized version of the Matrix Task^e^
Borella et al. (2017b)^f^	X		X		73	71.63 (5.53), 61–87	n.a.	9.42 (4.54)	Vocabulary score WAIS-R^c^, max. 70; 49.21 (10.89)	180 (three sessions of 60 min over 2 weeks) individual setting	Adaptive verbal working memory training with the CWMS^d^ Task *via* audio-recordings
Borella et al. (2017a)		X		X	54	Mozart 70.15 (2.79); Albinoni 69.31 (3.30); White noise 68.18 (3.48); 65–75	Mozart 11 ♀, 8 ♂; Albinoni 7 ♀, 12 ♂; White noise 12 ♀, 4 ♂	Mozart 13.84 (2.91); Albinoni 14.73 (2.15); White noise 13.06 (4.00)	n.a.	180 (three sessions of 60 min over 2 weeks) individual setting	6 min of listening to music according to experimental condition followed by adaptive verbal working memory training with the CWMS^d^ Task *via* audio-recordings
Borella et al. (2017c)	X		X	X	36	WM 69.44 (3.73); WM+S 67.94 (4.89)	WM 10 ♀, 8 ♂; WM+S 13 ♀, 5 ♂	WM 14.39 (2.87); WM+S 13.56 (2.92)	Vocabulary score WAIS-R^c^, max. 70; WM 61.72 (5.63); WM+S 58.39 (9.89)	105 (three sessions of 35 min over 2 weeks) individual setting	Adaptive verbal working memory training with the CWMS^d^ Task *via* audio-recordings; for the WM+S group preliminary instructions to use a visual mental imagery strategy^g^
Brehmer et al. (2011)		X	X	X	24	63.6 (SD n.a.); 60–70	12 ♀, 12 ♂	n.a.	n.a.	625 (25 sessions of 25 min over 5 weeks) home-based individual setting	Adaptive vs. non-adaptive (fixed at low level) both verbal and visuospatial working memory training with the computerized Cogmed^h^ training program
Brum et al. (2018)		X		X	41	Three sessions 67.17 (4.40); six sessions 67.91 (3.61)	n.a.	Three sessions 9.50 (5.25); six sessions 7.57 (3.34)	Clock Drawing Test^i^, max. 10; three sessions 9.00 (1.13); six sessions 8.83 (0.98)	Three sessions: 105 (three sessions of 35 min over 1 week) Six sessions: 210 (six sessions of 35 min over 2 weeks) individual setting	Adaptive verbal working memory training with the CWMS^d^ Task *via* audio-recordings
Heinzel et al. (2014a)	X	X	X		19	66.0 (3.73); 61–75	6 ♀, 13 ♂	15.61 (3.26)	n.a.	540 (12 sessions of 45 min over 4 weeks)individual setting	Adaptive computerized numerical n-back training paradigm^l^
Heinzel et al. (2014b)		X	X		25	Val/Val^j^ 67.36 (4.34); any Met 64.64 (3.37)	Val/Val 5 ♀, 6 ♂; any Met 7 ♀, 7 ♂	Val/Val 15.46 (3.15); any Met 16.88 (3.62)	MMSE^k^, max. 30; Val/Val 29.27 (1.01); any Met 29.64 (0.84)	540 (12 sessions of 45 min over 4 weeks) individual setting	Adaptive computerized numerical n-back training paradigm^l^
Matysiak et al. (2019)		X	X		43	65.9 (SD n.a.)	28 ♀, 15 ♂	n.a. Education dichotomized into higher (*n* = 27) *vs*. secondary (*n* = 16) education	operation span (OSPAN)^m^ score, max. n.a.; 15.31 (SD n.a.)	500 (25 sessions of 20 min over 5 weeks) home-based individual setting	Adaptive computerized dual (visuo-spatial and auditory/verbal) n-back training paradigm^n^
McAvinue et al. (2013)		X		X	19	69.89 (4.5); 64–79	13 ♀, 6 ♂	n.a. educational levels only: primary school *n* = 1; leaving certificate *n* = 2; undergraduate *n* = 10; postgraduate *n* = 6	MMSE^k^, max. 30; 27.74 (2.05); AMNART IQ^o\^, max. n.a.; 120.47 (4.44)	750 (25 sessions of 30 min over 5 weeks) home-based individual setting	Adaptive computerized verbal and visuo-spatial working memory training plus psychoeducation on everyday cognitive strategies
Simon et al. (2018)	X		X	X	82	Adaptive 72.4 (5.6); non-adaptive 73.7 (6.5)	Adaptive 29 ♀, 12 ♂; non-adaptive 26 ♀, 15 ♂	Adaptive 15.7 (3.7); non-adaptive 15.3 (3.2)	MMSE^k^, max. 30; adaptive 29.2 (1.1); non-adaptive 29.0 (1.3); AMNART IQ^o\^, max. n.a.; adaptive 122.6 (5.9); non-adaptive 120.6 (6.0)	1,000 (25 sessions of 40 min over 5 weeks) home-based individual setting	Adaptive *vs*. non-adaptive (fixed at low level) both verbal and visuospatial working memory training with the computerized Cogmed^h^ training program
Tusch et al. (2016)		X	X	X	41	Adaptive 74.47 (6.26); non-adaptive 76.84 (5.95)	Adaptive 12 ♀, 5 ♂; non-adaptive 15 ♀, 3 ♂	Adaptive 18.65(2.98); non-adaptive 16.78(2.05)	MMSE^k^, max. 30; adaptive 29.41 (0.71); non-adaptive 28.89 (1.68); AMNART AMNART IQ^o\^, max. n.a.; adaptive 123.59 (4.00); non-adaptive 119.33 (5.86) 119.33 (5.86)	1,000 (25 sessions of 40 min over 5 weeks) home-based individual setting	Adaptive *vs*. non-adaptive (fixed at low level) both verbal and visuospatial working memory training with the computerized Cogmed^h^ training program
Weicker et al. (2018)		X	X	X	40	Adaptive 67.8 (3.9); non-adaptive 67.7 (3.1) 67.7 (3.1)	Adaptive 10 ♀, 10 ♂; non-adaptive 11 ♀, 9 ♂	n.a. categorized only: <9 years *n* = 4; 10–12 years *n* = 16; >12 years *n* = 20	n.a.	540 (12 sessions of 45 min over 4 weeks) individual setting	Adaptive *vs*. non-adaptive (fixed at low-level) working memory training with the computerized WOME^p^ (WOrking MEmory) training program
Zinke et al. (2012)		X	X		20	86.8 (4.9); 77–96	14 ♀, 6 ♂	11.7 (3.3)	MMST short form for old-old adults^q^, max. 21; 19.4 (1.4)	275 (10 sessions of 25–30 min over 2 weeks) individual setting	Adaptive paper–pencil verbal and visuo-spatial working memory training
Zinke et al. (2014)	X		X		40	76.7 (8.4); 65–95	32 ♀, 8 ♂	14.4 (3.4)	MMST short form for old-old adults^q^, max. 21; 20.2 (1.1)	270 (nine sessions of 30 min over 3 weeks) individual setting	Adaptive paper–pencil verbal and visuo-spatial working memory and executive control training

a *Number of participants in the working memory training group*.

b *Young-old sample from Borella et al. (2010)*.

c *WAIS-R, Wechsler Adult Intelligence Scale-revised manual. Wechsler (1981)*.

d *CWMS, Categorization Working Memory Span. De Beni et al. (2008). Training procedure introduced by Borella et al. (2010)*.

e *Adapted from Cornoldi et al. (2007) and Carretti et al. (2012)*.

f *post-hoc analysis of Borella et al. (2010, 2013, 2017c); Carretti et al. (2013)*.

g *As described in Carretti et al. (2007)*.

h *For details about the adaptive training algorithm, see Cogmed QM; www.cogmed.com; Klingberg et al. (2002)*.

i *Aprahamian et al. (2010) and Shulman (2000)*.

j *Carriers of either Val/Met or Met/Met COMT (catechol-O-methyltransferase) genotype were classified into one group (any Met) and contrasted with Val/Val carriers*.

k *MMSE, Mini-Mental State Examination; Folstein et al. (2010)*.

l *Gevins and Gevins and Cutillo (1993)*.

m *Computerized version of the original OSPAN task; Turner and Engle (1989)*.

n *Introduced by Jaeggi et al. (2008)*.

o\ *AMNART, American National Adult Reading Test; Nelson (1982)*.

p *WOME, WOrking MEmory; part of the cognitive rehabilitation program RehaCom®*.

q *Mini-Mental State Examination short form for old-old adults by Kliegel et al. (2001)*.

### Reporting Quality and Risk of Bias

Table 2 reports the risk of bias per study across six domains evaluated with the QUIPS checklist (Hayden et al., 2013). A detailed risk of bias assessment on a single item level rather than QUIPS domain ratings can be obtained from the corresponding author. Important information is lacking throughout many of investigated studies, especially regarding the domains study attrition, study confounding, and statistical analysis and reporting. Most notably, the appropriate selection of the analysis plan and reporting of both the statistical analyses and results are often fragmentary. Only for the domains of prognostic factor measurement and outcome measurement were the majority of studies rated with low risk. In summary, the reporting quality was partly insufficient, and the results should be interpreted cautiously.

**Table 2:** Risk of bias assessment using the QUIPS checklist.

**References**	**Study participation**	**Study attrition**	**Prognostic factor measurement**	**Outcome measurement**	**Study confounding**	**Statistical analysis and reporting**
Borella et al. (2013)						
Borella et al. (2014)						
Borella et al. (2017b)						
Borella et al. (2017a)						
Borella et al. (2017c)						
Brehmer et al. (2011)						
Brum et al. (2018)						
Heinzel et al. (2014a)						
Heinzel et al. (2014b)						
Matysiak et al. (2019)						
McAvinue et al. (2013)						
Simon et al. (2018)						
Tusch et al. (2016)						
Weicker et al. (2018)						
Zinke et al. (2012)						
Zinke et al. (2014)						

*Overall risk of bias rating of domains in the Quality in Prognosis Studies (QUIPS) checklist (Hayden et al., 2013). Red, high risk; yellow, moderate risk; green, low risk. For details on individual items and rating scheme, please refer to Supplementary Material 3*.

Unfortunately, the initially planned meta-analysis could not be performed as the applied analytical approaches, as described below, were too heterogeneous and the reported results did not allow to compute comparable effect size measures (e.g., odds ratios, hazard ratios) across studies to meta-analyze the prognostic effects. Therefore, only a systematic review focusing on the directionality of prognostic effects rather than their magnitude was performed.

### Prediction Analyses and Outcome Measures

Seven of the 16 prognostic studies used more than one prediction analysis account to predict WMT responsiveness (one study included both a prediction model and a factor finding approach; six studies included more than one factor finding approach, i.e., investigated the prognostic value of one or several variables with at least two different approaches). Five studies investigated prediction models, three of which used hierarchical regression analyses (Heinzel et al., 2014a; Zinke et al., 2014; Borella et al., 2017c) with change scores or relative change scores as dependent variables. One study used Bayesian modeling approach (Borella et al., 2017b) and one used linear mixed effect modeling (Simon et al., 2018), both with time as one predictor, therefore abandoning the use of change scores as dependent variable. Ten studies were factor finding studies, including a total of 18 factor finding analysis approaches: seven used a generalized linear model approach (e.g., ANOVA) (Brehmer et al., 2011; Zinke et al., 2012; Borella et al., 2014; Heinzel et al., 2014b; Tusch et al., 2016; Brum et al., 2018; Weicker et al., 2018), one used ANCOVA (Borella et al., 2017a), five used Pearson correlations (Brehmer et al., 2011; Zinke et al., 2012; McAvinue et al., 2013; Heinzel et al., 2014a; Tusch et al., 2016), and one used linear regressions (Weicker et al., 2018) and one Linear Mixed Models (Matysiak et al., 2019). Three studies used a (descriptive) comparison of effect sizes (Borella et al., 2013, 2014; Brum et al., 2018). For the generalized mixed model approach, 71% used time as a predictor and only 29% used raw or standardized change scores as a dependent variable. For ANCOVA, the post-test score was used as a dependent variable. Pearson correlations and linear regressions used (standardized) change scores as dependent variables; for the linear mixed model, time was used as a predictor. None of the studies compared prognostic factors or models between the trained group and a passive control group, i.e., they analyzed the data of trained groups only. To summarize, even though prediction approaches were highly heterogeneous, analyses were comparable within the different approaches.

We defined verbal and visuospatial working memory, i.e., direct training and near-transfer effects, as primary outcomes. Most of the included studies distinguished between these two domains; however, four studies did not (Brehmer et al., 2011; Zinke et al., 2012; Simon et al., 2018; Weicker et al., 2018), and four studies addressed verbal working memory only (Heinzel et al., 2014a,b; Tusch et al., 2016; Matysiak et al., 2019). Three of the 16 included studies (18.8%) investigated direct training effects (i.e., effects in trained tasks) only (Heinzel et al., 2014a,b; Matysiak et al., 2019). The majority of studies (62.5%) investigated a combination of direct training, near-transfer (i.e., untrained working memory tasks), and several far-transfer measures, defined as secondary outcomes in our systematic review. Frequently investigated far-transfer cognitive domains were executive functions (including verbal fluency, reasoning, inhibition, set shifting, and executive control), processing speed (short-term), memory, and fluid intelligence. Only one study investigated non-cognitive outcomes (anxiety and depression) (McAvinue et al., 2013). Only three of the included studies (18.8%) did not apply a prognostic approach for at least one direct training outcome and instead focused on near- and far-transfer effects only (McAvinue et al., 2013; Tusch et al., 2016; Simon et al., 2018). Most studies used objective and standardized neuropsychological assessment tools. Others, for example, studies assessing (verbal) working memory by n-back tasks (25%), compared n-back task levels within different points of time or used indexes from signal detection theory (Heinzel et al., 2014a,b; Tusch et al., 2016; Matysiak et al., 2019) (for details on prediction analyses and outcomes, see Table 3 and Supplementary Material 4).

**Table 3:** Prognostic analyses, outcomes, results, and timing.

**Study**	**Analysis**				**Outcome**		**Prediction results**								**Timing**	
	**Model**	**Factor finding**				**Degree of transfer**	**Baseline performance**	**Intelligence**	**Age**	**Education**	**Sex**	**Adaptivity**	**Dose of training**	**Others**	**Post-intervention**	**Follow-up**
		**Corr/Reg**	**GLM**	**Others**												
Borella et al. (2013)				X effect size	Verbal working memory Δ*d*	+			↓						X	X 8
						^†^			↓						X	
					Visuospatial working memory Δ*d*	^†^			↓						X	
					Short-term memory Δ*d*	^‡^			↓						X	
					Fluid intelligence Δ*d*	^‡^			↓						X	
					Processing speed Δ*d*	^‡^			↓						X	
					Inhibition Δ*d*	^‡^			↓						X	
						^‡^			↓						X	
Borella et al. (2014)			X ANOVA		Verbal working memory Δ*s*	+			↓						X	X 8
					Visuospatial working memory Δ*s*	^†^			↓						X	X 8
				X effect size	Working memory Δ*d*	^†^								Training modality: visuospatial –	–	
					Verbal working memory Δ*d*	+								Training modality: visuospatial –	–	
					Visuospatial working memory Δ*d*	^†^								Training modality: visuospatial –	–	
					Short-term memory Δ*d*	^‡^								Training modality: visuospatial –	–	
					Fluid intelligence Δ*d*	^‡^								Training modality: visuospatial ↓	–	
					Processing speed Δ*d*	^‡^								Training modality: visuospatial –	–	
					Inhibition Δ*d*	^‡^								Training modality: visuospatial ↓	–	
Borella et al. (2017b)	X linear mixed models				Verbal working memory	+		↓							X	X 8
						^†^			↓	–					X	– 8
					Visuospatial working memory	^†^		↑	↓						X	– 8
					Short-term memory	^‡^	↓	↓		↓					X	– 8
					Fluid intelligence	^‡^	↓		↓						X	X 8
					Processing speed	^‡^	–	↑							X	– 8
					Inhibition	^‡^			↓						X	– 8
Borella et al. (2017a)			X ANOVA		Verbal working memory	+								Music listening condition: Albinoni ↑	X	– 6
					Visuospatial working memory	^†^								Music listening condition –	–	– 6
					Fluid intelligence	^‡^								Music listening condition: Albinoni ↑	X	– 6
					Phonemic verbal fluency	^‡^								Music listening condition –	–	– 6
Borella et al. (2017c)	X hierarchical regression				Verbal working memory Δ	+	↓							Strategy use ↑	X	
						^†^	↓									X 8
						^†^	↓									X 8
						^†^	↓									X 8
					Visuospatial working memory Δ	^†^	↓									X 8
					Processing speed Δ	^‡^	↓								X	
						^‡^	↓									X 8
Brehmer et al. (2011)			X ANOVA		Verbal working memory	^†^						–			–	
					Visuospatial working memory	^†^						↑			X	
					Short-term memory	^‡^						–			–	
						^‡^						–			–	
					Episodic memory	^‡^						↑			X	
					Attention	^‡^						↑			X	
					Reasoning	^‡^						–			–	
					Inhibition	^‡^						–			–	
		X Pearson			Working memory Δmax	+	↑^*^								X	
Brum et al. (2018)			X ANOVA		Verbal working memory	+							–		–	– 6
						^†^							–		–	– 6
						^†^							–		–	– 6
					Visuospatial working memory	^†^							–		–	– 6
						^†^							–		–	– 6
					Verbal short-term memory	^‡^							–		–	– 6
					Visuospatial short-term memory	^‡^							–		–	– 6
					Reasoning	^‡^							–		–	– 6
					Inhibition	^‡^							–		–	– 6
					Semantic fluency	^‡^							–		–	– 6
				X Effect size	Verbal working memory Δ*d*	+							–		–	– 6
						^†^							↑		–	X 6
						^†^							↓		X	X 6
					Visuospatial working memory Δ*d*	^†^							↓		–	X 6
						^†^							–		–	– 6
					Verbal short-term memory Δ*d*	^‡^							–		–	– 6
					Visuospatial short-term memory Δ*d*	^‡^							↓		–	X 6
					Reasoning Δ*d*	^‡^							↑		X	X 6
					Inhibition Δ*d*	^‡^							↑		X	X 6
					Semantic fluency Δ*d*	^‡^							↓		–	X 6
Heinzel et al. (2014a)	X hierarchical regression				Verbal working memory Δ	+			↑	↓	?			Baseline load-dependent BOLD ↑ gray matter volume ↑	X	
						+	↑		?	?	?			Baseline load-dependent BOLD ↑ gray matter volume?	X	
		X Pearson			Verbal working memory Δ	+								Baseline load-dependent BOLD ↑	X	
Heinzel et al. (2014b)			X ANOVA		Verbal working memory	+								Val/Val ↓	X	
Matysiak et al. (2019)		X linear mixed models			Verbal working memory max	+	↑^*^									
						+			–							
						+				–						
						+					–					
						+								Occupational activity –		
McAvinue et al. (2013)		X Pearson			Short-term memory	^‡^							↓		X	
						^‡^							–		–	
					Long-term memory	^‡^							–		–	
					Anxiety and depression	^‡^							↓		X	
Simon et al. (2018)	X linear mixed models				Working memory	^†^						↑		Processing speed?	X	
						^†^			–			↑			X	
					Processing speed	^‡^			–			–			–	
					Set shifting	^‡^			–			–			–	
					Phonemic fluency	^‡^			–			–			–	
					Semantic fluency	^‡^			–			–			–	
Tusch et al. (2016)			X ANOVA		Verbal working memory	^†^						–			–	
		X Pearson			Verbal working memory Δ	^†^		–							–	
						^†^				–					–	
Weicker et al. (2018)			X ANOVA		Working memory	^†^						–			–	– 3
					Working memory span	^†^						↑			X	– 3
					Visuospatial working memory	^†^						↑			X	– 3
					Executive functions	^‡^						–			–	– 3
					Logical reasoning	^‡^						–			–	– 3
					Long-term memory	^‡^						–			–	– 3
		X Pearson			Working memory Δmax	+	↑^*^								X	
						+	↑^*^								X	
						+	↑^*^								X	
Zinke et al. (2012)			X *t*-tests		Working memory Δ	+	↓								X	
		X Pearson			Verbal working memory Δ	^†^	↓								X	
					Visuospatial working memory Δ	^†^	↓								X	
						^†^	↓								X	
					Verbal short-term memory Δ	^‡^	↓								X	
					Visuospatial short-term memory Δ	^‡^	↓								X	
Zinke et al. (2014)	X hierarchical regression				Verbal working memory Δ	+	↓	–	↓						X	
						^†^		–	–					Training task gains ↑	X	
					Visuospatial working memory Δ	+	↓	–	↓						X	
						^†^		–	↓					Training task gains –	X	
					Executive control Δ	+	↓	↑	–						X	
						^†^		–	↓					Training task gains ↓↑	X	
					Fluid intelligence Δ	^‡^		–	↑					Training task gains –	X	
					Inhibition Δ	^‡^		↑	↓					Training task gains –	X	

*Detailed information on the analytical plan of each prediction approach and the operationalization of both prognostic variables and outcomes; extracted results can be obtained from Supplementary Material 4 and Supplementary Material 5. If not indicated otherwise, time was included as one factor in the analyses, therefore abandoning the use of change scores as dependent variable and investigating more than one point of time (e.g., pre-test, post-test, and follow-up) within one analysis. Within prediction results, ↑ indicates positive predictors, i.e., higher values in the predictor variable are associated with better training outcomes, ↓ indicates negative predictors, i.e., lower values in the predictor variable are associated with better training outcomes; – indicates non-significant relationships between predictor and training outcome, and?indicates that a predictor was investigated, but results were not reported appropriately. For prognostic model studies, only the final models reported in the original manuscript are reported. In the Timing column, an X indicates a significant influence of the prognostic factor(s) under investigation on the respective outcome at the given point of time; – non-significant relationships only. For follow-ups, time in months is indicated*.

*Δ, unstandardized/raw change score as dependent variable; Δd, Cohen's d as dependent variable; Δmax, maximum change score; Δs, standardized change score as dependent variable [(post–pre)/SD_pre_]; Corr/Reg, analytical approaches including correlations, linear regressions, multi-level modeling approaches; GLM, generalized linear model approaches including ANOVA, ANCOVA, independent sample t-tests*.

* *Dependent variable represents the maximum level/change achieved during training*.

+ *Direct training effect, i.e., task was trained within the working memory training*.

† *Near-transfer effect, i.e., task was not trained within the working memory training but represents (verbal and/or visuospatial) working memory*.

‡ *far-transfer effect, i.e., task was not trained within the working memory training and does not represent (verbal and/or visuospatial) working memory*.

### Predictor Variables and Prediction Results

Several different predictors for WMT responsiveness were investigated, including individual-related sociodemographic factors (e.g., age, sex, education), cognitive variables (baseline performance, intelligence, processing speed), and biological factors (genes, brain metabolism) as well as training-related factors (e.g., adaptivity, dose of training). There were 13 analysis approaches that investigated individual-related prognostic factors only, two analysis approaches investigated a combination of individual- and training-related characteristics, and eight analysis approaches investigated training-related characteristics only as predictors for WMT responsiveness. The results of the prognostic analyses are reported in Table 3. As in most cases, the direction of predictor effects did not vary systematically between single-outcome variables, and within prognostic factor finding vs. prognostic model studies, we decided to not further distinguish between different outcome variables and prognostic factor finding vs. prognostic model studies but indicate if prognostic effects were found for direct training and/or near- and/or far-transfer effects only. The described patterns of prognostic effects only reflect frequencies of observed prognostic relationships and do not take into account risk of bias and further methodological shortcomings of the underlying studies.

Age was investigated in four of five prognostic model studies and three of 18 factor finding approaches. With only few exceptions for single-outcome measures reporting positive or non-significant relationships, age was consistently found to be a negative predictor for WMT responsiveness across direct training as well as both near- and far-transfer effects, i.e., younger participants benefited more from the training than older participants independent of outcome measures. Note, however, that age as a continuous variable was dichotomized into young-olds *vs*. old-olds for three analytical approaches investigating age as a predictor for WMT responsiveness (Borella et al., 2013, 2014; Simon et al., 2018).

Education was investigated within two prognostic model and two factor finding approaches. Education most frequently constituted a negative predictor for direct training as well as near- and far-transfer effects (Heinzel et al., 2014a; Borella et al., 2017b); however, some analyses do not yield a significant relationship at all (Tusch et al., 2016; Matysiak et al., 2019). Whereas education was treated as a continuous variable in most studies, Matysiak et al. (2019) dichotomized the variable for their analysis. Sex was investigated in one prognostic model and one factor finding approach but was not found to be a significant predictor of WMT responsiveness in direct training effects (Heinzel et al., 2014a; Matysiak et al., 2019) and was not investigated in any prognostic approach on near- and/or far-transfer measures.

Baseline performance in working memory tasks and/or outcome measures was the most frequently investigated prognostic factor (four of five prognostic model studies and five of 18 factor finding approaches). For both near- and far-transfer outcomes, baseline working memory and/or baseline performance in outcome measure was consistently found to be a negative predictor for WMT responsiveness (Zinke et al., 2012; Borella et al., 2017b,c), i.e., individuals with lower performance at baseline improved more from WMT than individuals with higher baseline performance. However, for analyses on direct training effects, heterogeneous results appear, with some analyses indicating baseline working memory and/or baseline performance in outcome measure to be a positive predictor (Brehmer et al., 2011; Heinzel et al., 2014a; Weicker et al., 2018; Matysiak et al., 2019), i.e., individuals with higher baseline performance in training tasks achieving higher WMT task gains than individuals with lower baseline performance. Baseline performance, as a continuous variable, was dichotomized into high *vs*. low performers by median split in two of the analytical approaches (Zinke et al., 2012; Matysiak et al., 2019).

Intelligence was investigated within two of five prognostic model studies and one of 18 factor finding approaches. For direct transfer effects, the prognostic value remains unclear (Zinke et al., 2014; Borella et al., 2017b). Furthermore, whereas there does not seem to be a significant predictive value when intelligence is investigated as a prognostic factor for near-transfer effects (Zinke et al., 2014; Tusch et al., 2016) or when evidence points to different prognostic directions (Borella et al., 2017b), a different pattern emerges for far-transfer effects: if significant, for the majority of far-transfer effect outcomes, the analyses indicate intelligence to be a positive predictor of gains after WMT (Zinke et al., 2014; Borella et al., 2017b), i.e., individuals with higher intelligence show larger far-transfer effects after targeted WMT than individuals with lower intelligence.

In the only study (prognostic model and prognostic factor) investigating a functional imaging parameter as predictor for WMT gains, individuals with a blood oxygen level-dependent (BOLD) response pattern more similar to younger adults (i.e., higher load-dependent network Delta scores) showed higher direct WMT gains (Heinzel et al., 2014a). Only one study investigated a genetic factor, yielding carriers of the Val/Val catechol-O-methyltransferase (COMT) genotype to show less direct training effects after WMT than the carriers of any Met COMT genotype (Heinzel et al., 2014b).

With regard to training-related prognostic factors, the prognostic effects of dose of training (investigated within two studies) were mixed for both near- and far-transfer effects (McAvinue et al., 2013; Brum et al., 2018), only marginally comparable between studies because of different prognostic factor operationalizations and not investigated for direct training effects. Adaptivity was investigated within four studies and, if significant, showed to be a positive predictor for WMT responsiveness (Brehmer et al., 2011; Simon et al., 2018; Weicker et al., 2018), with adaptive training regimes yielding better results than non-adaptive training regimes, especially for near-transfer effects.

## Discussion

This systematic review is the first one to evaluate prognostic factors and models for WMT responsiveness in healthy older adults. Within the 16 studies meeting our inclusion criteria, five prognostic model approaches and 18 factor finding approaches were included. One of the main findings is that the methodological and reporting quality of prognostic research within the evaluation of WMT regimes in healthy older adults is often insufficient; therefore, no meta-analysis could be conducted and no clear conclusions can be drawn from the systematic review. Age, education, intelligence, and baseline performance in working memory or other cognitive domains were frequently investigated predictors across studies. However, given the methodological shortcomings of the included studies, emerging patterns of prognostic effects across direct training as well as near- and far-transfer effects will have to survive sound methodological replication in future attempts to promote precision medicine approaches in the context of WMT.

First, our findings will be discussed within the methodological framework of prognostic research; secondly, they will be related to the theoretical framework of cognitive aging and embedded into other prognostic research literature in the field of cognitive interventions, and thirdly, they will be linked to findings from a prognostic review on memory trainings in healthy older adults (Roheger et al., 2020).

### Methodological Considerations

Several methodological considerations and implications can be derived from the present systematic review. First of all, it has confirmed that prognostic research in the area of WMT in healthy older adults is not yet fully established and is rather premature. The prognostic framework is usually not indexed, and the specific mention of the prognostic approach in titles or abstracts is limited as well (Riley et al., 2019). For example, within our included studies, only five studies used a prediction-related terminology in their titles (Heinzel et al., 2014a,b; Zinke et al., 2014; Borella et al., 2017b; Matysiak et al., 2019).

Furthermore, large heterogeneity appears throughout the included studies with regard to study design (e.g., randomized controlled trials vs. cohort studies vs. *post hoc* analyses) and the applied analytical approaches. The applied analytical approaches did not only differ widely *per se* but have differing suitability to answer the question “who benefits most” from WMT regimes in healthy older adults. In general, a prognostic factor is defined as any measure that, among people with a given condition, is associated with a subsequent outcome (Riley et al., 2013), therefore implying at least some kind of a causal relationship. The majority of studies in our systematic review, however, used group comparisons (e.g., by ANOVA, *t*-test, comparison of effect sizes) to investigate the influence of a group characteristic on a given outcome. Despite the fact that these approaches can only state whether the compared groups differ from one another and not whether the investigated group characteristic linearly correlate with or even causally predict the investigated outcome, another important point needs to be highlighted: Whereas some investigated prognostic factors are innately categorical (e.g., sex, training modality, adaptivity), originally continuous predictors (e.g., age, baseline performance) were frequently dichotomized into artificial groups, for example, young-olds vs. old-olds (Borella et al., 2013, 2014; Simon et al., 2018) and high vs. low performers (Zinke et al., 2012; Matysiak et al., 2019). Dichotomization of both dependent and independent variables is strongly discouraged as it results in loss of information, possible misunderstandings of actual continuous relationships, and severe loss of power (Dawson and Weiss, 2012; Moreau et al., 2016; Fernandes et al., 2019).

Another frequently used analytical approach was the computation of correlation coefficients between predictor variables and change scores in outcome measures after WMT. However, no causal interferences can be derived from correlation analyses (Bewick et al., 2003). Furthermore, correlations, for example, between baseline performance and change scores (which is obtained by subtracting baseline performance from post-training performance), are less more than pure statistical artifacts (Smoleń et al., 2018). Smoleń et al. (2018) discuss that, unfortunately, even more advanced methods such as multiple regressions and linear mixed models do not guarantee the correct assessment of relationships between predictor variables and respective outcomes. According to the authors, the only correct method would be to use direct modeling of correlations between latent true measures and gain by structural equation modeling (Smoleń et al., 2018). Future research on prognostic factors regarding (working memory) training responsiveness should apply advanced statistical methods such as latent difference score models or growth curve analyses as highly flexible statistical approaches from the structural equation modeling background. On the one hand, this would allow to circumvent several statistical fallacies clinical trial data often include, such as violations of multivariate normality assumptions, non-linear change trajectories, and missing data patterns (Newsom, 2015). On the other hand, it would allow to explore the (statistical) properties of change through training without actually calculating change scores and with highly flexible options to model interdependencies between several variables (Smoleń et al., 2018).

In this context, one immense problem arises within prognostic research on cognitive intervention programs *per se* and WMT in particular: the lack of statistical power due to small sample sizes. Prognostic research requires large sample sizes, with a representative distribution of individuals' characteristics and values across the prognostic factors of interest. Especially for (cognitive) training studies, researchers are confronted with the challenge to overcome a self-selection bias to not only engage highly educated, active, and motivated individuals within their trials (Oswald et al., 2006; Schubert et al., 2014). As prognostic research in this field often arises as an (explorative) add-on or *post hoc* analysis of former data from randomized controlled trials, sample size calculations at the stage of study design (if present at all) do only take into account the sample size needed to evaluate the effectiveness of a training regime (by comparing the experimental group against at least one control group). For future research in the field of personalized prevention and treatment approaches for healthy aging, we encourage to emphasize the outstanding importance of prognostic research by focusing on the prognostic aim already during study design.

Importantly, as already discussed in the introduction, prognostic analyses should always include data of at least one control group as well to detangle predictors of specific treatment response from general prognostic factors of retest effects such as practice effects and regression to the mean (Hingorani et al., 2013). None of the studies included in this systematic review followed this recommendation. Therefore, the identified prognostic relationships might represent systematic relationships; however, they might exist in both treated and untreated individuals and, therefore, not represent true predictors of treatment response.

Beyond that, however, the large body of data on WMT effectiveness for healthy older adults bears the enormous potential of *post hoc* prognostic analyses, for example, as executed by Borella et al. (2017b). Within the tradition of evaluating similar WMT regimes, over the years several randomized controlled trials to investigate the efficacy of similar training regimes were carried out in this study group. As Borella et al. (2017b) recognized large variability in the effectiveness of WMT across individuals on the one hand and large heterogeneity across results on earlier investigations on the influence of individual characteristics on training outcomes on the other hand, they merged the data of four earlier training studies (Borella et al., 2010, 2013, 2017c; Carretti et al., 2013) to investigate an individual's characteristics related to WMT gains in a larger sample. In other words, they conducted a tiny-scale individual participant data (IPD) meta-analysis, the gold standard for meta-analytical approaches. At this point, it should be noted that Borella et al. (2017b) included data of participants from the training groups of Borella et al. (2013) and Borella et al. (2017c), two studies included in our systematic review as well. Therefore, the prognostic results of these three studies are not fully independent. However, we did not exclude the two earlier works, as the exclusion would not have changed the results on the (qualitative) directional prognostic effects. For future IPD meta-analysis, the IPD data of either the four mentioned studies or Borella et al. (2017b) should be included only.

Regarding the analytical approaches used and the results of this review, it should further be mentioned that the recommendation to focus on adjusted results to reveal whether a certain index factor contributes independently and above other prognostic factors (Riley et al., 2019) could not be met entirely: most of the included studies in this review investigated only one prognostic factor per analysis. However, as established prognostic factors did not (yet) exist in the context of WMT responsiveness, analytical approaches excluding possibly important confounding variables are (at least in parts) comprehensible as well. For future prognostic research in this field, however, we recommend to include baseline performance and age as a minimum set of control variables when investigating further prognostic factors.

### Prognostic Factors for Working Memory Training Responsiveness

Several different predictors for WMT responsiveness were investigated, including individual-related sociodemographic factors (e.g., age, sex, education), cognitive variables (baseline performance, intelligence), biological factors (brain metabolism, genes) as well as training-related factors (e.g., adaptivity, dose of training). Given the methodological shortcomings of the included studies discussed above, no clear conclusions regarding prognostic effects can be drawn. Emerging patterns based on frequently observed prognostic effects will have to survive sound methodological replication in future attempts to promote precision medicine approaches in the context of WMT. Some inconsistent findings might be due to statistical and psychometric artifacts, uncontrolled extraneous influences, or the absence of convincing robust prognostic relationships at all. Nevertheless, we would like to provide a contextual framework for the discussion of possible predictors for WMT responsiveness beyond pure methodological issues.

The most frequently investigated predictor was baseline performance. Despite the many different statistical approaches and poor reporting quality in most studies, baseline performance was, with exceptions for direct training effects only (Brehmer et al., 2011; Heinzel et al., 2014a; Weicker et al., 2018; Matysiak et al., 2019), identified as a negative predictor, i.e., individuals with lower baseline performance are the ones that benefit most from WMT in terms of performance on neuropsychological tests in the domains of working memory and other cognitive functions (e.g., executive functions, short-term memory). Therefore, most inconsistencies regarding the directionality of the prognostic effect of baseline performance could be elucidated when taking a look at the operationalization of the dependent variables. The finding of baseline performance being a negative predictor for cognitive intervention responsiveness is also common for targeted memory trainings (Roheger et al., 2020) as well as other cognitive intervention approaches such as multidomain cognitive trainings (Whitlock et al., 2012; López-Higes et al., 2018; Roheger et al., 2019). However, opposing findings exist as well, indicating that higher baseline performance might be indicative for cognitive intervention success (Fairchild et al., 2013; Willis and Caskie, 2013). However, given the lack of comparisons of prognostic factors between WMT and control groups within the included studies, the frequently observed negative associations between baseline performance and change through training might simply represent effects of regression to the mean (Smoleń et al., 2018). This statistical artifact causes negative correlations between baseline performance and gain by noisy repeated measurements, where extreme values at the first point of time tend to be closer to the mean at the second point of time, without reflecting a real change (Smoleń et al., 2018).

Nevertheless, baseline performance as a predictor for training responsiveness can be discussed within the compensation vs. magnification framework (Lövdén et al., 2010, 2012). Following this account, individuals with lower baseline performance would show higher training benefits because they have more room for improvement, whereas individuals with higher baseline performance already perform at ceiling, leaving less room for improvement. Improvements across individuals performing less optimal at baseline might therefore represent some kind of flexibility rather than plasticity. According to Lövdén et al. (2010), flexibility represents “the capacity to optimize the brain's performance within current structural constraints, using the available range of existing representational states.” Beyond this flexibility, plasticity denotes the capacity for extending the range of representational states, where flexibility then operates. This understanding of plasticity, however, fits better with the magnification hypothesis, constituting that individuals with higher cognitive abilities would benefit most, as they have more resources “to acquire, implement, and sharpen effortful cognitive strategies” (Lövdén et al., 2012).

Within our systematic review, we also found hints for this dualism between compensation vs. magnification or rather flexibility vs. plasticity. Whereas, our findings regarding baseline performance in neuropsychological test measures might rather reflect mechanisms following the compensation account, our findings regarding age as a possibly negative predictor and intelligence as a possibly positive predictor for WMT responsiveness are more interpretable in terms of the magnification account. Higher (crystallized) intelligence might constitute the required “hardware” to utilize the possibilities given by WMT to extend the cognitive repertoire and, in the broadest sense, reflecting cognitive plasticity. This perspective is strengthened considering our finding that intelligence seems to be a positive predictor for gains after WMT for far-transfer effects only. Whereas, lower baseline performance might be predictive for both near- and far-transfer effects (interpreted in terms of the compensation account and flexibility: if there is room for improvement, performance will be optimized by training), higher cognitive abilities might be especially beneficial for far-transfer effects, i.e., to transfer direct training effects to untrained cognitive domains. The magnification account might additionally be able to explain our finding that baseline performance in trained tasks sometimes emerged as a positive predictor for direct training effects. As most WMT regimes adapted their difficulty to user performance across the course of training and no ceiling effects could be expected, higher initial levels might represent general cognitive ability rather than task-specific baseline, and participants with higher initial levels in training tasks might be more able to utilize the whole potential of the training regime.

The second most frequently investigated predictor was age, indicating that older individuals might benefit less from WMT than younger individuals, even within the cohort of healthy older adults above the age of 55. Age might be a proxy for the course of the interplay between neural and cognitive plasticity, which yields a higher potential for plastic changes in younger age than in old-old age (Burke and Barnes, 2006; Greenwood and Parasuraman, 2010; Li, 2013). Due to age-related reductions in processing resources (Park and Bischof, 2013; Paraskevoudi et al., 2018), the ability to engage in plastic changes after WMT might be reduced in older age. This was already reflected in an early meta-analysis on moderators of memory training effects (Verhaeghen et al., 1992). However, findings in contemporary cognitive intervention literature diverge and either report no significant relationship (Willis and Caskie, 2013; Roheger et al., 2019), positive relationships (i.e., the older the individual, the more benefits) (Brooks et al., 1999), or negative relationships (i.e., the younger the individual, the more benefits) (Fairchild et al., 2013). In terms of differential prognostic effects for different training regimes (e.g., WMT vs. memory training), this will be further discussed below.

The only study investigating brain imaging parameters as predictors for WMT responsiveness strengthens the finding of our systematic review that age might be a negative predictor for positive training responsiveness: Heinzel et al. (2014a) found a more “youth-like” BOLD response pattern in healthy older adults to be predictive of increased working memory performance after training. This youth-like response pattern is reflected in a higher load-dependent working memory network Delta score, indicating that both high working memory network efficiency (represented by decreased activation during low-level tasks) and high working memory network capacity (represented by increased activation during high-level tasks) are related to plasticity (Barulli and Stern, 2013). This BOLD response pattern has also been discussed as a biomarker for cognitive reserve (Stern, 2009). Against this backdrop, one could hypothesize that cognitive reserve and brain reserve constitute higher-order predictors for WMT success and are operationalized by several different proxies within the existing prognostic research approaches (Stern et al., 2018).

Within the cognitive reserve framework, it is not uncommon to find education alone as a proxy for this construct (Stern, 2002; Valenzuela and Sachdev, 2006; Stern et al., 2018). In our systematic review, we found a tendency of education being a negative predictor of WMT responsiveness. In cognitive intervention research, it is discussed that cognitive interventions might be able to diminish the cognitive reserve disadvantage of less-educated older adults (Clark et al., 2016; Mondini et al., 2016), thereby leading to more training-related gains. As this might appear counterintuitive at first, it is important here to differentiate between brain reserve and lifetime proxies of cognitive reserve such as education, occupational attainment, and leisure time activities (Stern et al., 2019). A higher cognitive reserve is commonly associated with less cognitive deficits, given the same brain pathology (Wilson et al., 2013; Hoenig et al., 2019). It follows that two individuals with similar cognitive functioning but different educational backgrounds might also differ in their brain pathology, i.e., the individual with higher education might already show a higher level of brain pathology compared to the individual with lower education, which in turn comes down to lower levels of brain reserve for individuals with higher education. Therefore, for the individual with lower education, even though the lifetime cognitive reserve is lower, the brain reserve might be higher, which corresponds to a better hardware to adapt training benefits.

Only one study investigated a genetic factor as predictor for WMT responsiveness in healthy older adults (Heinzel et al., 2014b), revealing carriers of the Val/Val COMT genotype, which is associated with reduced prefrontal dopamine metabolism, to benefit less from WMT than carriers of any Met COMT genotype. The COMT genotype affects prefrontal dopamine metabolism, which is itself related to cognitive plasticity (higher prefrontal dopamine metabolism = more cognitive plasticity) (Frias et al., 2005; Diamond, 2007). Furthermore, previous research indicated that advantageous dopamine-related genes are critically involved in working memory performance and the ability to benefit from WMT (Brehmer et al., 2009; Bellander et al., 2011; Bäckman and Nyberg, 2013), which further strengthens the finding of Heinzel et al. (2014b) that these relationships are also present in healthy older adults.

We did not find a consistent influence of sex on responsiveness to WMT in healthy older adults, even though some kind of “sex-specific plasticity” and following sex-specific differences between training responsiveness to different cognitive domains are proposed in literature (Beinhoff et al., 2008; Rahe et al., 2015; Roheger et al., 2019). Note, however, that sex as a prognostic factor for WMT responsiveness was investigated in two studies with direct training effects as dependent variable only. Therefore, no final conclusions can be drawn. Even though motivational factors and personality traits are discussed to play a significant role in predicting responsiveness to general cognitive interventions (Colquitt et al., 2000; West et al., 2008; Studer-Luethi et al., 2012; Double and Birney, 2016; Kalbe et al., 2018), they were not yet investigated as prognostic factors within the WMT context.

Summarizing possible prerequisites for WMT responsiveness, we hypothesize that there has to be not only room for improvement (i.e., lower baseline performance) to engage in training-related cognitive flexibility but also sufficient “hardware” (e.g., age, intelligence, brain metabolism, genetic variation) to engage in training-related cognitive and neural plasticity. It needs to be highlighted again that the body of evidence (so far) is too weak to draw clear conclusions. Even though some findings fit well into the compensation vs. magnification account and the cognitive reserve framework, future studies of high methodological quality will have to replicate those findings.

Regarding dose of training as one training-related prognostic factor investigated in the context of WMT responsiveness, results were mixed and are in accordance with heterogeneous results in literature. For example, Teixeira-Santos et al. (2019) identified shorter compared to longer training durations to be beneficial for training outcome. However, they discuss this finding to be unexpected and influenced by confounding factors such as the type of outcome variable and highly heterogeneous training durations that impede comparability between studies. All of the included studies in our review implemented an adaptive training regime, where the task difficulty adapted to user performance. Four studies compared adaptive vs. non-adaptive WMT regimes, with adaptivity emerging as a positive predictor for training responsiveness. Adaptivity of trained task difficulty is discussed to contribute to the maintenance of training motivation and the avoidance of underchallenging and overstraining participants during training (Weicker et al., 2016). However, some studies did not find beneficial effects of implementing individually adaptive training regimes (von Bastian and Eschen, 2016).

Only one study within our systematic review used a multi-domain training. Zinke et al. (2014) included an executive control task next to several working memory tasks within their WMT regime. Executive control might, however, strongly be dependent on working memory (Chai et al., 2018). Even though we cannot evaluate the contribution of single training tasks or the training of single domains to the overall prognostic effects, we conclude that this exception from targeted WMT does not constitute a danger for the validity of our findings regarding WMT responsiveness.

### Working Memory Training vs. Memory Training

Just recently, a systematic review on prognostic factors of memory improvements after memory training using a similar systematic review technique has been published (Roheger et al., 2020). Roheger et al. (2020) identified further methodological shortcomings of prognostic research in the context of memory training and, on a content-related level, more vulnerable individuals (e.g., lower baseline performance, higher age) to benefit most from memory training. They also identified several “hardware” factors (e.g., hippocampal volume, genetic variation in apolipoprotein-E-4) as prognostic factors. Primarily, however, the direction of age as a prognostic factor seems to differ between the two training regimes.

We hypothesize this difference to be due to the different cognitive training approaches investigated. Memory training, as investigated by Roheger et al. (2020), can be referred to as a strategy-based training, whereas WMT can be referred to as a process-based training (Lustig et al., 2009; Teixeira-Santos et al., 2019). Whereas strategy-based trainings focus on the application of specific strategies to a task where the target population typically does poorly, process-based trainings focus on tasks that load on a specific cognitive function, however, without explicit strategy training (Lustig et al., 2009). Thereby, process-based trainings are believed to produce more transfer effects to untrained domains, as untrained cognitive functions might depend on the targeted cognitive domain (Lustig et al., 2009; Teixeira-Santos et al., 2019). This difference in the conceptualization of memory training vs. WMTs, however, implicates different levels of cognitive demands that have to be met in order to benefit from the trainings. Given the higher cognitive demands of WMT, we hypothesize that younger individuals might benefit more, as their hardware potential to engage in neural and cognitive plasticity is higher. Older individuals, however, might be less able to engage in neural plasticity but might therefore rather benefit from strategy-based training approaches, optimizing their cognitive performance within a given structural constraint in terms of flexibility (Lövdén et al., 2010, 2012). In the framework of Lövdén et al. (2012), WMT gains equal practice gains that are related to plasticity and better fit the magnification model, whereas memory training gains equal instruction gains that are related to flexibility and better fit the compensation account. Further research is necessary to prove this concept, but we are convinced that these findings highlight the urgent need for personalized cognitive prevention and intervention methods to counteract cognitive decline at best for every individual.

Another systematic review and meta-analysis on prognostic factors and models of cognitive and behavioral changes after multidomain cognitive training in healthy older adults is still ongoing (preliminary Prospero ID 147531). Those findings, in combination with the findings of the present systematic review and of Roheger et al. (2020), will further contribute to the understanding of which cognitive interventions yield best outcomes for which individual. Furthermore, the discussion around precision medicine in the context of cognitive interventions can be taken to a whole new level if one would not only consider the cognitive domain trained (or the combination of domains) but also the nature of the training tasks, the training setting (e.g., computerized *vs*. paper–pencil *vs*. mixed, home-based *vs*. individual *vs*. group settings), and its intensity. So far, however, the body of data is too small for subgroup analyses.

### Strengths and Limitations

This systematic review is, to the authors' best knowledge, the first one to systematically assess prognostic factors and models for WMT responsiveness in healthy older adults on a single-person-within-study level rather than investigating moderating factors in a meta-analysis on a study-wide aggregated level as done in a recent meta-analysis on WMT in healthy older adults (Teixeira-Santos et al., 2019). Further strengths include the applied methods following the PICOTS system to define our review question, the CHARMS checklist for data extraction, and the PRISMA guidelines for the reporting of systematic reviews (Moher et al., 2009; Moons et al., 2014; Debray et al., 2017; Riley et al., 2019). One limitation is that, due to insufficient reporting quality throughout many of the included studies, the studies in their entirety were sometimes difficult to comprehend, information might be misinterpreted by the reviewers, and results should be interpreted cautiously. It follows that as already discussed above, due to methodological heterogeneity, we were not able to perform a quantitative meta-analysis but had to focus on the qualitative directionality of the prognostic effects, limiting the validity of our findings. Furthermore, the applied WMT regimes within our included studies were highly heterogeneous regarding training duration, training tasks, and training setting. Only a multi-level IPD meta-analysis might be able to appropriately investigate the interplay of training-related and individual characteristics to answer the question “who benefits most.” Additionally, the analyses to identify predictors of WMT responsiveness were conducted with data of the WMT groups only. Therefore, they did not control for effects in the control group (Hingorani et al., 2013), which impedes disentangling predictors of WMT responsiveness from predictors of retest and practice effects (Calamia et al., 2012). In this context, we need to admit that on the design stage of this systematic review, no comparator factor (C in PICOTS) was being considered as our aim was to systematically assess any approach to prognostic research on WMT responsiveness. Furthermore, even though the risk of bias assessment followed the QUIPS checklist (Hayden et al., 2013) across six domains, the overall rating procedure across the items of one domain and across the six domains is not standardized by the developers.

## Conclusion

To summarize, prognostic research within the evaluation of WMT regimes in healthy older adults is still underrepresented given the urgent need for personalized cognitive prevention and intervention methods to counteract cognitive decline. Given the methodological shortcomings of the included studies, no clear conclusions can be drawn, and emerging patterns of prognostic effects will have to survive sound methodological replication in future attempts to promote precision medicine approaches in the context of WMT. However, within the small body of evidence and despite the complex relationships between cognitive reserve, neural plasticity, and different proxies for these constructs, it seems that the requirements for both, flexibility and plasticity, have to be met. An IPD meta-analysis might be able to overcome the current research gaps regarding prognostic factors for WMT responsiveness in healthy older adults.

## Data Availability Statement

The original contributions presented in the study are included in the article/Supplementary Material, further inquiries can be directed to the corresponding author/s.

## Author Contributions

AO, MR, and EK conceptualized the presented work. MR conducted the systematic search. NS contributed to the systematic search. AO, MR, and A-KF conducted the title and abstract screening. AO and MR conducted the full text screening, extracted the data, and conducted the risk of bias assessment. AO drafted the first version of the manuscript. EK supervised the project during each stage of work. All authors revised the manuscript for intellectual content and approved the final version of the manuscript.

## Conflict of Interest

AO reports no conflicts of interest. MR has received a grant from the Brandau-Laibach Stiftung, and a grant from the German Ministry of Education and Research. A-KF has received a grant from the German Parkinson Society, and honoraria from ProLog Wissen GmbH, Cologne, Germany, pro audito Switzerland, Zürich, Switzerland, Seminar- und Fortbildungszentrum Rheine, Germany, and LOGOMANIA, Fendt & Sax GbR, Munich, Germany. A-KF was author of the cognitive training program NEUROvitalis but receives no corresponding honoraria. EK has received grants from the German Ministry of Education and Research, ParkinsonFonds Deutschland gGmbH, the German Parkinson Society and honoraria from Oticon GmbH, Hamburg, Germany, Lilly Pharma GmbH, Bad Homburg, Germany, Bernafon AG, Bern, Switzerland, and Desitin GmbH, Hamburg, Germany. EK was author of the cognitive training program NEUROvitalis but receives no corresponding honoraria. The remaining author declares that the research was conducted in the absence of any commercial or financial relationships that could be construed as a potential conflict of interest.
