# Supplementary material for: A Systematic Review on Predictors of Working Memory Training Responsiveness in Healthy Older Adults: Methodological Challenges and Future Directions
Source: Front Aging Neurosci. 2020 Oct 14;12:575804. doi: 10.3389/fnagi.2020.575804 (PMC7591761; doi:10.3389/fnagi.2020.575804)
Supplement: Supplementary file 1 [file Data_Sheet_1.PDF]

Supplementary Material

A Systematic Review on Predictors of Working Memory Training Responsiveness in Healthy Older Adults: Methodological Challenges and Future Directions

1 PRISMA checklists

1.1 PRISMA Checklist for Abstracts

1.2 PRISMA Checklist for Systematic Reviews

2 Systematic Search Strategy: CENTRAL, Medline, PsycInfo, Web of Science Core Collection

3 Risk of Bias Assessment Using the Quality in Prognosis Studies (QUIPS) checklist

4 Outcomes, Prognostic Factors and Details on Analyses of the Included studies

5 Overview of Prognostic Results

1 PRISMA checklists

1.1 PRISMA Checklist for Abstracts

Section/topic	Checklist item	Reported on page #
1. Title:	Identify the report as a systematic review, meta-analysis, or both.	1
**BACKGROUND**		
2. Objectives:	The research question including components such as participants, interventions, comparators, and outcomes.	1
**METHODS**		
3. Eligibility criteria:	Study and report characteristics used as criteria for inclusion.	1
4. Information sources:	Key databases searched and search dates.	1
5. Risk of bias:	Methods of assessing risk of bias.	1
**RESULTS**		
6. Included studies:	Number and type of included studies and participants and relevant characteristics of studies.	1
7. Synthesis of results:	Results for main outcomes (benefits and harms), preferably indicating the number of studies and participants for each. If meta-analysis was done, include summary measures and confidence intervals.	1
8. Description of the effect:	Direction of the effect (i.e. which group is favoured) and size of the effect in terms meaningful to clinicians and patients.	1
**DISCUSSION**		
9. Strengths and Limitations of evidence:	Brief summary of strengths and limitations of evidence (e.g. inconsistency, imprecision, indirectness, or risk of bias, other supporting or conflicting evidence)	1-2
10. Interpretation:	General interpretation of the results and important implications	1-2
**OTHER**		
11. Funding:	Primary source of funding for the review.	2
12. Registration:	Registration number and registry name.	2

1.2 PRISMA Checklist for Systematic Reviews

Section/topic	#	Checklist item	Reported on page #
**TITLE**			
Title	1	Identify the report as a systematic review, meta-analysis, or both.	1
**ABSTRACT**			
Structured summary	2	Provide a structured summary including, as applicable: background; objectives; data sources; study eligibility criteria, participants, and interventions; study appraisal and synthesis methods; results; limitations; conclusions and implications of key findings; systematic review registration number.	1-2
**INTRODUCTION**			
Rationale	3	Describe the rationale for the review in the context of what is already known.	2-3
Objectives	4	Provide an explicit statement of questions being addressed with reference to participants, interventions, comparisons, outcomes, and study design (PICOS).	3
**METHODS**			
Protocol and registration	5	Indicate if a review protocol exists, if and where it can be accessed (e.g., Web address), and, if available, provide registration information including registration number.	3
Eligibility criteria	6	Specify study characteristics (e.g., PICOS, length of follow-up) and report characteristics (e.g., years considered, language, publication status) used as criteria for eligibility, giving rationale.	4
Information sources	7	Describe all information sources (e.g., databases with dates of coverage, contact with study authors to identify additional studies) in the search and date last searched.	3-4
Search	8	Present full electronic search strategy for at least one database, including any limits used, such that it could be repeated.	Suppleme ntary Material 2
Study selection	9	State the process for selecting studies (i.e., screening, eligibility, included in systematic review, and, if applicable, included in the meta-analysis).	4
Data collection process	10	Describe method of data extraction from reports (e.g., piloted forms, independently, in duplicate) and any processes for obtaining and confirming data from investigators.	4
Data items	11	List and define all variables for which data were sought (e.g., PICOS, funding sources) and any assumptions and simplifications made.	
Risk of bias in individual studies	12	Describe methods used for assessing risk of bias of individual studies (including specification of whether this was done at the study or outcome level), and how this information is to be used in any data synthesis.	4, Suppleme ntary Material 3
Summary measures	13	State the principal summary measures (e.g., risk ratio, difference in means).	4-5
Synthesis of results	14	Describe the methods of handling data and combining results of studies, if done, including measures of consistency (e.g., I^2) for each meta-analysis.	4-5
Risk of bias across studies	15	Specify any assessment of risk of bias that may affect the cumulative evidence (e.g., publication bias, selective reporting within studies).	4-5
Additional analyses	16	Describe methods of additional analyses (e.g., sensitivity or subgroup analyses, meta-regression), if done, indicating which were pre-specified.	n.a.

**RESULTS**			
Study selection	17	Give numbers of studies screened, assessed for eligibility, and included in the review, with reasons for exclusions at each stage, ideally with a flow diagram.	6, Figure 1
Study characteristics	18	For each study, present characteristics for which data were extracted (e.g., study size, PICOS, follow-up period) and provide the citations.	6 Table 1
Risk of bias within studies	19	Present data on risk of bias of each study and, if available, any outcome level assessment (see item 12).	6, Table 2
Results of individual studies	20	For all outcomes considered (benefits or harms), present, for each study: (a) simple summary data for each intervention group (b) effect estimates and confidence intervals, ideally with a forest plot.	6-16, Table 3
Synthesis of results	21	Present results of each meta-analysis done, including confidence intervals and measures of consistency.	n.a.
Risk of bias across studies	22	Present results of any assessment of risk of bias across studies (see Item 15).	n.a.
Additional analysis	23	Give results of additional analyses, if done (e.g., sensitivity or subgroup analyses, meta-regression [see Item 16]).	n.a.
**DISCUSSION**			
Summary of evidence	24	Summarize the main findings including the strength of evidence for each main outcome; consider their relevance to key groups (e.g., healthcare providers, users, and policy makers).	16
Limitations	25	Discuss limitations at study and outcome level (e.g., risk of bias), and at review-level (e.g., incomplete retrieval of identified research, reporting bias).	16-20
Conclusions	26	Provide a general interpretation of the results in the context of other evidence, and implications for future research.	16-20
**FUNDING**			
Funding	27	Describe sources of funding for the systematic review and other support (e.g., supply of data); role of funders for the systematic review.	21

From: Moher D, Liberati A, Tetzlaff J, Altman DG, The PRISMA Group (2009). Preferred Reporting Items for Systematic Reviews and Meta-Analyses: The PRISMA Statement. PLoS Med 6(7): e1000097. doi:10.1371/journal.pmed1000097

2 Systematic Search Strategy: CENTRAL, Medline, PsycInfo, Web of Science Core Collection)

CENTRAL
1. "healthy older adults": ti, ab, kw
2. "healthy elderly": ti, ab, kw
3. MeSH descriptor: [Healthy aging]
4. "older adults": ti, ab, kw
5. MeSH descriptor: [Aged]
6. MeSH descriptor: [Aged, 80 and over"]
7. "elderly individuals": ti, ab, kw
8. "cognitive aging": ti, ab, kw
9. "cognitive intervention": ti, ab, kw
10. "cognitive training": ti, ab, kw
11. "brain training": ti, ab, kw
12. "memory training": ti, ab, kw
13. "reasoning training": ti, ab, kw
14. "mnemonic training": ti, ab, kw
15. "training": ti, ab, kw
16. "intervention": ti, ab, kw
17. MeSH descriptor: [Memory]
18. "memory": ti, ab, kw
19. {or #1-#8}
20. {or #9-#16}
21. #17 or #18
22. #19 and #20 and #21

Access: 01.10.2019
Results: 1380

PsycInfo
1. exp memory/
2. exp Aging/
3. "healthy older adults" .mp.
4. "healthy elderly" .mp.
5. "older adults" .mp.
6. "cognitive aging" .mp.
7. "aged (attitudes toward)"/
8. "cognitive intervention" .mp.
9. "cognitive training" .mp.
10. "brain training" .mp.
11. "memory training" .mp.
12. "reasoning training" .mp.
13. "mnemonic training" .mp.
14. "training" .mp.
15. "intervention" .mp.
16. exp brain stimulation/ or exp brain training/
17. 8 or 9 or 10 or 11 or 12 or 13 or 14 or 15 or 16
18. 2 or 3 or 4 or 5 or 6 or 7
19. 1 and 17 and 18

Access: 01.10.2019
Results: 829

Medline
1. "healthy older adults" [All fields]
2. "healthy elderly" [All fields]
3. "Healthy aging" [MeSh]
4. "older adults" [All fields]
5. "Aged" [Mesh:NoExp]
6. "Aged, 80 and over" [MeSh]
7. "elderly individuals" [All fields]
8. "cognitive aging" [All fields]
9. 1 OR 2 OR 3 OR 4 OR 5 OR 6 OR 7 OR 8
10. "cognitive intervention" [All fields]
11. "cognitive training" [All fields]
12. "brain training" [All fields]
13. "memory training" [All fields]
14. "reasoning training" [All fields]
15. "mnemonic training" [All fields]
16. "training" [All fields]
17. "intervention" [All fields]
18. 10 OR 11 OR 12 OR 13 OR 14 OR 15 OR 16 OR 17
19. "memory" [MeSh]
20. "memory" [all fields]
21. 19 OR 20
22. 9 AND 18 AND 21

Access: 01.10.2019
Results: 3335

Web of Science Core Collection

#5 #4 AND #3 AND # 2
Indexes=SCI-EXPANDED, SSCI, A&HCI, ESCI
Timespan=All years

#4 TS = memory
Indexes=SCI-EXPANDED, SSCI, A&HCI, ESCI
Timespan=All years

#3 TS = ("cognitive intervention" OR "cognitive training" OR "brain training" OR "memory training" OR "reasoning training" OR "mnemonic training" OR "training" OR "intervention")
Indexes=SCI-EXPANDED, SSCI, A&HCI, ESCI
Timespan=All years

#2 TS = ("healthy older adults" OR "healthy elderly" OR "Healthy aging OR "older adults" OR Aged OR Aged, 80 and over OR "elderly individuals" OR "cognitive aging")
Indexes=SCI-EXPANDED, SSCI, A&HCI, ESCI
Timespan=All years

Access: 01.10.2019
Results: 7422

3 Risk of Bias Assessment Using the Quality in Prognosis Studies (QUIPS) checklist

Domains and subdomains assessed with the QUIPS tool	Issues to consider according to the QUIPS tool and judgment reasons of the review authors
**Study participation**	The domain was rated with high risk, if no inclusion or exclusion criteria were stated, or if more than two subdomains were rated as "high risk". It was rated as "moderate risk", if two domains were rated with a "high risk".
- Source of target population	The source population or population of interest is adequately described.
- Method used to identify population	The sampling frame and recruitment are adequately described, including methods to identify the sample sufficient to limit potential bias
- Recruitment period	Period of recruitment was described.
- Place of recruitment	Place of recruitment (setting and geographic location) are adequately described
- Inclusion and exclusion criteria	Inclusion and exclusion criteria are adequately described (e.g., including explicit diagnostic criteria or "zero time" description).
- Adequate study participation	There is adequate participation in the study by eligible individuals
- Baseline characteristics	The baseline study sample (i.e., individuals entering the study) is adequately described for at least the variables age, sex, and education.
**Study Attrition**	The domain was rated with high risk, if more than two subdomains were rated as "high risk". It was rated as "moderate risk", if two domains were rated with a "high risk" or if either "Reasons for lost to follow-up" or "Outcome and prognostic factor information" was rated with a "high risk".
- Proportion of baseline sample available for analysis	Response rate (i.e., proportion of study sample completing the study and providing outcome data) is adequate.
- Attempts to collect information on participants who dropped out	Attempts to collect information on participants who dropped out of the study are described.
- Reasons and potential impact of subjects lost to follow-up	Reasons for loss to follow-up are provided.
- Outcome and prognostic factor information on those lost to follow-up	Participants lost to follow-up are adequately described, for at least age, sex, and education.<br>There are no important differences between key characteristics (age, sex, education) and outcomes in participants who completed the study and those who did not.
**Prognostic Factor Measurement**	The domain was rated with high risk, if more than two subdomains were rated as "high risk" or if the subdomain "definition of prognostic factor" was rated as "high risk". It was rated as "moderate risk", if two domains were rated with a "high risk".
- Definition of the PF	A clear definition or description of 'PF' is provided.
- Valid and Reliable Measurement of PF	Method of PF measurement is adequately valid and reliable to limit misclassification bias.<br>Continuous variables are reported or appropriate cut-points (i.e., not data-dependent) are used.
- Method and Setting of PF Measurement	The method and setting of measurement of PF is the same for all study participants.
- Proportion of data on PF available for analysis	Adequate proportion of the study sample has complete data for PF variable.
- Method used for missing data	Appropriate methods of imputation are used for missing 'PF' data.
**Outcome Measurement**	The domain was rated with "high risk", if one subdomain was rated as "high risk".

- Definition of the Outcome	A clear definition of outcome is provided, including duration of follow-up and level and extent of the outcome construct.
- Valid and Reliable Measurement of Outcome	The method of outcome measurement used is adequately valid and reliable to limit misclassification bias.
- Method and Setting of Outcome Measurement	The method and setting of outcome measurement is the same for all study participants.
**Study Confounding**	The domain was rated with “high risk”, if two or more subdomains were rated as “high risk”. The domain was rated with “medium risk” if one domain was rated with “high risk”.
- Important Confounders Measured	Important confounders, including treatments, are measured.
- Definition of the confounding factor	Clear definitions of the important confounders measured are provided.
- Valid and Reliable Measurement of Confounders	Measurement of all important confounders is adequately valid and reliable (e.g., may include relevant outside sources of information on measurement properties, also characteristics, such as blind measurement and limited reliance on recall).
- Method and Setting of Confounding Measurement	The method and setting of confounding measurement are the same for all study participants.
- Method used for missing data	Appropriate methods are used if imputation is used for missing confounder data
- Appropriate Accounting for Confounding	Important potential confounders are accounted for in the study design or in the analysis.
**Statistical Analysis and reporting**	The domain was rated with “high risk” if the subdomain “Presentation of analytical strategy” was rated as “high risk” or if more than one of the other subdomains was rated with “high risk”. The domain was rated with “medium risk” if one subdomain (except the first) was rated with “high risk”.
- Presentation of analytical strategy	There is sufficient presentation of data to assess the adequacy of the analysis.
- Model development strategy	The strategy for model building (i.e., inclusion of variables in the statistical model) is appropriate and is based on a conceptual framework or model.<br>The selected statistical model is adequate for the design of the study (e.g. regression model, mixed models).
- Reporting of results	There is no selective reporting of results.

Nach Hayden, J. A., van der Windt, D. A., Cartwright, J. L., Côté, P., & Bombardier, C. (2013). Assessing bias in studies of prognostic factors. *Annals of Internal Medicine, 158*(4), 280-286.

4 Outcomes, Prognostic Factors and Details on Analyses of the Included studies

Study	Outcomes			Prognostic Factors			Analysis	
	Operationalization	Handling in analysis	Blinding?	Operationalization	Handling in analysis	Blinding?	Method	Assumptions checked?
Borella, Carretti, Zanoni, Zavagnin, and De Beni (2013)	**Verbal Working Memory** by CWMS Criterion Task, Backward Digit Span; **Visuospatial Working Memory** by Dot Matrix; **Short-term memory** by Forward Digit Span; **Fluid Intelligence** by Cattell test; **Inhibition** by Stroop Color test interference index (RT); **Processing Speed** by Pattern comparison	standardized gain score/ effect size Cohen's d for difference Δpost-pre and Δfollow-up-pre	n.a.	**age**	dichotomized into young-old vs. old-old	n.a.	Descriptive comparison of effect sizes (Cohen's d) between age groups	n.a.
Borella et al. (2014)	**Verbal Working Memory** by CWMS Criterion Task; **Visuospatial Working Memory** by Dot Matrix	standardized gain scores for short-term gains ([individual posttest score – individual pretest score]/pretest *SD*) and for maintenance gains ([individual follow-up score – individual pretest score]/pretest *SD*).	n.a.	**age**	dichotomized into young-old vs. old-old	n.a.	2x2 ANOVA with age-group (young-old vs. old-old) and training group (WMT group vs. CG) as between-subject variables. Post-hoc comparison by t-tests.	n.a.
	**Working Memory** by Backward Digit Span / CORSI block span; **Verbal Working Memory** by CWMS Criterion Task; **Visuospatial Working Memory** by Dot Matrix; **Short-term memory** by Forward Digit Span / CORSI block span; **Fluid Intelligence** by Cattell test; **Inhibition** by Stroop Color test interference index (RT); **Processing Speed** by Pattern comparison	standardized gain score/ effect size Cohen's d for difference Δpost-pre	n.a.	**training modality**	categorical (verbal vs. visuospatial)	n.a.	Descriptive comparison of effect sizes (Cohen's d) for young-olds between training modalities	n.a.
Borella, Carbone, Pastore, De Beni, and Carretti (2017)	**Verbal Working Memory** by CWMS Task; **Visuospatial Working Memory** by Dot Matrix; **Short-term memory** by Forward Digit Span; **Verbal Working Memory** by Backward Digit Span; **Fluid Intelligence** by Cattell test; **Processing Speed** by Pattern Comparison;	time as independent variable in the analysis (baseline, post-test, follow-up)	n.a.	**age** in years; **education** in years; **intelligence** crystallized intelligence by WAIS-R vocabulary score; **baseline working memory** by baseline CWMS performance	continuous	n.a.	Linear Mixed Models were fitted separately for each outcome measure for each combination of predictors using the Bayesian MCMC estimation method. Comparisons between the models were based on the Widely Applicable Information Criterion (WAIC; Watanabe, 2010), and the AkaikeWeight. The best model was analyzed using posterior distributions of parameters.	graphical inspection of the univariate and bivariate distributions of all the outcome variables considered, then their

	**Inhibition** by Stroop Color test interference index (RT)						Parameter estimates were summarized by using posterior means and 95% Credibility Intervals.	summarizing with descriptive statistics.
Borella, Carretti, Meneghetti, et al. (2017)	**Verbal Working Memory** by CWMS Criterion Task; **Visuospatial Working Memory** by Backward CORSI span; **Phonemic Verbal Fluency; Fluid Intelligence** by Culture Fair test; **Spatial Visualization** by Minnesota Paper Form Board; **Spatial Learning** by Spatial descriptions - map drawings	the post-test score or the follow-up score as dependent variables, the pre-test score as a covariate	n.a.	**music listening condition** (Mozart's Sonata K448, Albinoni's Adagio in G minor, White Noise)	categorical	n.a.	Univariate ANCOVAs using the post-test score or the follow-up score as dependent variables, the pre-test score as a covariate, and music listening condition as a between-subjects factor. Helmert contrast were used to compare the white noise condition vs the music conditions (Mozart; Albinoni), and finally the two different music conditions (Mozart vs Albinoni).	n.a.
Borella, Carretti, Sciore, et al. (2017)	**Verbal Working Memory** by CWMS Task, Listening Span, and Backward Digit Span; **Visuospatial Working Memory** by Jigsaw Puzzle; **Processing Speed** by Pattern Comparison	change scores; short-term training gains as difference Δpost-pre; long-term training gains as difference Δfu-pre	yes	**baseline performance** in outcome measure; **training gains** (immediate or long-term) in the criterion task (CWMS); **strategy use** (dummy coded, 0 = without, 1 = with)	continuous for baseline performance and training gains; categorical for strategy use	yes	Hierarchical regression models for each outcome in which training effects were found.<br>1st step: baseline performance in outcome measure<br>2nd step: gains (immediate or long-term) in the CWMS<br>3rd step: strategy use (dummy coded group variable).<br>Best model was selected based on explained variance.	n.a.
Brehmer et al. (2011)	**Verbal working memory** by Digit Span Backward; **Visuospatial Working Memory** by Span Board Backward; **Short-term memory** by Span Board Forward and Digit Span Forward; **Attention** by PASAT; **Reasoning** by RAVEN; **Episodic memory** by RAVLT; **Inhibition** by Stroop Color Interference	time as independent variable in the analysis (baseline, post-test)	n.a.	**adaptivity**	categorical (adaptive vs. non-adaptive)	n.a.	ANOVAs with group (adaptive training vs. active control) as a between-subjects factor and time (baseline vs. post training) as a within-subjects factor	n.a.
	**Working Memory** by scores in Cogmed WM training	maximum gain score as maximum score that individuals reached during Cogmed WM training minus baseline performance	n.a.	**baseline working memory** as mean of the first two training sessions	continuous	n.a.	Pearson correlation between baseline performance and maximum gain score	n.a.
Brum, Borella, Carretti, and Sanches Yassuda (2018)	**Verbal Working Memory** by CWMS, Digit Span Backward, and Letter Number Sequence; **Visuospatial working memory** by Spatial Span Backward and Symbol Search; **Short-term memory** by Digit Span Forward, Spatial Span Forward; **Semantic Verbal Fluency**; **Reasoning** by Matrix Reasoning; **Inhibition** by Stroop Color Interference	time as independent variable in the analysis (baseline, post-test, follow-up)	yes	**dose of training**	dichotomized (3 vs. 6 sessions)	yes	Repeated measures ANOVAs (3x2x2) with Time (Pre Test x Posttest x Follow up) as a within subjects factor and Condition (Trained group x Control group) and Dose (3 sessions x 6 sessions of training) as between subjects factors	n.a.

	**Verbal Working Memory** by CWMS, Digit Span Backward, and Letter Number Sequence; **Visuospatial working memory** by Spatial Span Backward and Symbol Search; **Short-term memory** by Digit Span Forward, Spatial Span Forward; **Semantic Verbal Fluency**; **Reasoning** by Matrix Reasoning; **Inhibition** by Stroop Color Interference	standardized gain score/ effect size Cohen's d for difference Δpost-pre and Δfollow-up-pre	yes	**dose of training**	dichotomized (3 vs. 6 sessions)	yes	Cohen's d effect sizes were transformed into r values and three and six session r values were quantitatively compared	n.a.
Heinzel, Lorenz, et al. (2014)	**Verbal Working Memory** by relative n-back task	standardized change scores; difference of the mean *n*-back performance at posttest and pretest (Δpost-pre) divided by performance at pretest	n.a.	**baseline load-dependent BOLD response pattern of working memory network** by working memory network Delta score**:** difference between the parameter estimates of the 3-back versus 0-back contrast and the parameter estimates of the 1-back versus 0-back contrast; **baseline working memory** by baseline n-back performance; **gray matter volume; age** in years; **sex**; **education** in years	continuous except for sex	n.a.	Hierarchical regression models with gray matter volume, age, sex and education as covariates. Evaluation of additional variance explained by baseline load-dependent BOLD response pattern of working memory network, and baseline working memory at pretest	n.a.
	**Verbal Working Memory** by relative n-back task	standardized change scores; difference of the mean *n*-back performance at posttest and pretest (Δpost-pre) divided by performance at pretest	n.a.	**baseline load-dependent BOLD response pattern of working memory network**	continuous	n.a.	Pearson correlation between baseline load-dependent BOLD response pattern of working memory network and verbal working memory short-term training gains.	n.a.
Heinzel, Riemer, et al. (2014)	**Verbal Working Memory Training performance** by n-back task level (Task difficulty was adaptively increased by reducing the interstimulus interval and by increasing the memory load from 2-back up to 5-back)	time as independent variable in the analysis	n.a.	**COMT genotype**	categorical (Val/Val vs. any Met)	n.a.	A repeated measures ANOVA (2 × 12) with genotype as between subjects factor and training session as within subject factor	n.a.
Matysiak, Kroemeke, and Brzezicka (2019)	**Verbal Working Memory Training Performance** by maximum n-back-level achieved during each training session	time as independent variable in the analysis	n.a.	**age** in years; **sex; education occupational activity**; **baseline working memory**	continuous for age, categorical for sex and occupational activity (active vs. retired); dichotomized	n.a.	Multilevel modeling (MLM) with repeated measurements nested within participants. Both fixed (the regression intercept and slope for the average person) and random effects (between-subject variability around the average) were examined. In Model 1, the change in N-back task scores over time was modeled represented by the number of a training session as a predictor (time). To test predicting and moderating effects of	n.a.

					for education (by level: higher vs. secondary) and baseline working memory (by baseline OSPAN score dichotomized to high vs. low)		demographics (age, sex, education level, occupational activity) as well as the influence of a baseline OSPAN score (between-person predictors – level 2) on within-subject variation (level 1) in N-back training additional models (2 to 6) were calculated. To avoid multicollinearity, all predictors were tested separately. The restricted maximum likelihood (REML) was used as the estimator. The first-order autoregressive covariance structure was used for the models, given the common proximal autocorrelation in the daily data	
McAvinue et al. (2013)	**Verbal Short-term memory** by Digit Span Forwards**; Verbal Short-term memory** by Word Recall Immediate; **Verbal Long-term memory** by Word Recall Delayed; **Hospital Anxiety and Depression Scale Post-Training Score**	standardized change scores; difference Δpost-pre divided by performance at pretest	n.a.	**time spent training**	continuous	n.a.	Pearson correlations between time spent training and proportional improvement during Post-Training Assessment ((Post-Training Assessment Score - Pre-Training Assessment Score)/Pre-Training Assessment Score)	n.a.
Simon et al. (2018)	**Working Memory** by Digit Symbol test; **Processing Speed** by TMT-A; **Set shifting** by by TMT-B; **Phonemic Fluency** by COWAT; **Semantic Verbal Fluency**	time as independent variable in the analysis (baseline, post-test)	yes	**adaptivity**; **speed of processing** (by TMT-A); **age**	continuous for speed of processing; dichotomized for adaptivity (adaptive vs. non-adaptive) and age (young-old vs. old-old by median split)	yes	Linear Mixed Model (LMM) included the fixed main effects of the intervention condition, (adaptive versus non-adaptive), assessment time (pre versus post), and processing speed or age group (young-old vs. old-old), as well as all possible two-way interactions and the three-way interaction between intervention condition, time and age. The models included participants as random intercepts to adjust for within-participant correlations of repeated measures. The parameters were estimated using the Restricted Maximum Likelihood Method, and unstructured covariance was specified to model the covariance structure of both the residuals and the random factors. To reduce false discovery rate (FDR), p-values for the interactions were adjusted employing the Benjamini-Hochberg (BH) procedure.	n.a.
Tusch et al. (2016)	**Verbal Working Memory** by A' discrimination index for n-back stimuli Variable from signal detection theory as speed/accuracy trade-off in processing and diminishing the influence of strategy effects	time as independent variable in the analysis (baseline, post-test)	n.a.	**adaptivity**	categorical (adaptive vs. non-adaptive)	n.a.	A repeated measures ANOVA (2 x 3 x 2) with time (pre and post) and n-back level (0-back, 1-back, 2-back) as within subject factors and adaptivity as between subject factor	n.a.
	**Verbal Working Memory** by A' discrimination index for n-back stimuli Variable from signal detection theory as speed/accuracy trade-off in processing and diminishing the influence of strategy effects	change score by difference Δpost-pre of A' discrimination index	n.a.	**education** in years; **intelligence** by AMNART IQ	continuous	n.a.	Pearsson correlations between A’ difference scores and education and intelligence.	n.a.
Weicker et al. (2018)	**Working memory functioning** by composite of span tasks, spatial addition, symbol span, n-back task,	time as independent variable in the	yes	**adaptivity**	categorical (adaptive vs. non-adaptive)	yes	Repeated measures ANOVAs (3 x 2) with the adaptivity (adaptive, non-adaptive, and control) as between subjects factor and time (pre vs. post or	n.a.

	PASAT, OSPAN; **Working memory span** by composite of Span Board and Digit Span; **Visuospatial Working Memory** by Span Board backward; **Executive Functions** composite of Stroop, Trail Making Test and TAP mental flexibility; **Logical resoning** by Leistungsprüfsystem Subtest 3; **Long-term verbal memory** by Rex Auditory Verbal Learning test	analysis (baseline vs. post-test; or baseline vs. follow-up)					pre vs. follow-up) as within-subjects factor. Post-hoc t-tests with Bonferroni correction to compare effects of adaptivity between training groups.	
	**Working Memory** by training task	Change score by difference of Δpost-pre of maximal level of training task	n.a.	**baseline working memory** by maximally attained level at baseline; **baseline verbal working memory** by baseline performance in digit span backward; **baseline visuo-spatial working memory** by baseline performance in spatial addition	continuous	n.a.	n.a.<br>not adequately reported. Presumably linear regressions.	n.a.
Zinke, Zeintl, Eschen, Herzog, and Kliegel (2012)	**Working memory**	change scores; short-term training gains as difference Δpost-pre	n.a.	**baseline working memory**	dichotomized (high- vs. low-capacity by median split on pre-training performance in each training task)	n.a.	Independent sample t-tests on working memory training gains between high- and low-capacity individuals	n.a.
	**Verbal working memory** by digit span backward**; Visuospatial working memory** by CORSI block span backward and K-ABC; **Verbal short-term memory** by digit span forward; **Visuospatial Short-term memory** by CORSI block span forward	change scores; short-term training gains as difference Δpost-pre	n.a.	**baseline performance** / **baseline working memory** capacity in each of the trained tasks	continuous	n.a.	Pearson correlations between baseline working memory capacity and training gains in each training task	n.a.
Zinke et al. (2014)	**Verbal Working Memory** by subtract-2-span task and Letter-span plus; **Visuospatial Working Memory** by (modified) K–ABC spatial memory task and by CORSI block span; **Executive Control** by Tower of London task and Tower of Hanoi; **Fluid Intelligence** by Raven Standard Progressive Matrices; **Inhibition** by Stroop interference	change scores; short-term training gains as difference Δpost-pre	n.a.	**age** in years; **intelligence** crystallized intelligence by the German Vocabulary test MWT-B; **baseline working memory** for prediction of gains in training task; **training task gains** (verbal working memory, visuospatial working memory, executive control) for prediction of far transfer gains	continuous	n.a.	Hierarchical regression analyses for each training gain<br>1st step: age<br>2nd step: crystallized intelligence<br>3rd step for training gains: baseline performance in the respective training<br>3rd step for near and far transfer tasks: gains in each of the trained tasks	n.a.

Note. For references of operationalization tools / neuropsychological assessments for outcomes and prognostic factors, consult original manuscripts.

5 Overview of Prognostic Results

Study	Unadjusted Results	Adjusted Results	Set of adjusted factors used	Non-linear relation?	Modelling assumptions?
Borella et al. (2013)	**Verbal Working Memory** by CWMS Criterion Task: age ↓ (short-term: d = 2.25 vs. d = 1.40; long-term: d = 2.01 vs. d = 1.44); by Backward Digit Span: age ↓ (short-term: d = 2.35 vs. d = 0.09); **Visuospatial Working Memory** by Dot Matrix: age ↓ (short-term: d = 1.70 vs. d = 0.47); **Short-term memory** by Forward Digit Span: age ↓ (short-term: d = 2.24 vs. d = 1.07); **Fluid Intelligence** by Cattell test: age ↓ (short-term: d = 1.40 vs. d = 0.15); **Inhibition** by Stroop Color test interference index (RT): age ↓ (short-term: d = 0.85 vs. d = -0.61); **Processing Speed** by Pattern comparison: age ↓ (short-term: d = 0.99 vs. d = 0.24)	n.a.	no adjustment	n.a.	n.a.
Borella et al. (2014)	**Verbal Working Memory** by CWMS Criterion Task: age ↓ (short-term and long-term); **Visuospatial Working Memory** by Dot Matrix: age – (short-term and long-term)	n.a.	no adjustment	n.a.	n.a.
	**Verbal Working Memory** by CWMS Criterion Task: training modality: visuospatial WM -- (short-term: d = 2.25 vs. d = 2.25); **Visuospatial Working Memory** by Dot Matrix: training modality: visuospatial WM -- (short-term: d = 1.70 vs. d = 2.1); **Short-term memory** by Forward Digit Span / CORSI block span: training modality: visuospatial WM -- (short-term: d = 2.24 vs. d = 1.55); **Working Memory** by Backward Digit Span / CORSI block span: training modality: visuospatial WM -- (short-term: d = 2.35 vs. d = 1.9); **Fluid Intelligence** by Cattell test: training modality: visuospatial WM ↓ (short-term: d = 1.40 vs. d = 0.45); **Inhibition** by Stroop Color test interference index (RT): training modality: visuospatial WM ↓ (short-term: d = 0.85 vs. d = 0.2); **Processing Speed** by Pattern comparison: training modality: visuospatial WM -- (short-term: d = 0.99 vs. d = 1.35)	n.a.	no adjustment	n.a.	n.a.
Borella, Carbone, et al. (2017)	n.a.	**Verbal Working Memory** by CWMS Task: intelligence ↓ (short-term, maintained at follow-up), by Backward Digit Span: age ↓ (short-term only) * education --; **Visuospatial Working Memory** by Dot Matrix: age ↓ * intelligence ↑ (both short-term only); **Short-term memory** by Forward Digit Span: education ↓ * intelligence ↓ * baseline working memory ↓ (all short-term only); **Fluid Intelligence** by Cattell test: age ↓ (short-term, maintained at follow-up) * baseline working memory ↓ (short-term only); **Processing Speed** by Pattern Comparison: intelligence ↑ (short-term only) * baseline working memory --; **Inhibition** by Stroop: age ↓ (short-term only)	Varied per model. Only best models were reported.	no	yes
Borella, Carretti, Meneghetti, et al. (2017)	**Verbal Working Memory** by CWMS Criterion Task: music – (short-term and long-term), Albinoni ↑ (short-term only); **Visuospatial Working Memory** by Backward CORSI span: music – (short-term and long-term), Albinoni – (short-term and long-term); **Phonemic Verbal Fluency** music – (short-term and long-term), Albinoni – (short-term and long-term); **Spatial Visualization** by Minnesota Paper Form Board: music – (short-term and long-term), Albinoni – (short-term and long-term); **Spatial Learning** by Spatial	n.a.	no adjustment	n.a.	n.a.

	descriptions - map drawings: music – (short-term and long-term), Albinoni – (short-term and long-term); **Fluid Intelligence** by Culture Fair test: music – (short-term and long-term), Albinoni ↑ (short-term only)				
Borella, Carretti, Sciore, et al. (2017)	**Verbal Working Memory – long-term training gains** by CWMS Task Δfu-pre: baseline performance ↓ (β = -0.37), by Listening Span Δfu-pre: baseline performance ↓ (β = -0.62), by Backward Digit Span Δfu-pre: baseline performance ↓ (β = -0.36); **Visuospatial Working Memory – long term training gains** by Jigsaw Puzzle Δfu-pre: baseline performance ↓ (β = -0.53); **Processing Speed – short-term training gains** by Pattern Comparison Δpost-pre: baseline performance ↓ (β = -0.84); **long-term training gains** by Pattern Comparison Δfu-pre: baseline performance ↓ (β = -0.66)	**Verbal Working Memory – short-term training gains** by CWMS Task Δpost-pre: baseline performance ↓ (β = -0.38) * strategy use ↑ (β = 0.32)	Varied per model. Only best models were reported.	no	yes
Brehmer et al. (2011)	**Verbal working memory** by Digit Span Backward: adaptivity –; **Visuospatial Working Memory** by Span Board Backward: adaptivity ↑; **Short-term memory** by Span Board Forward: adaptivity –; by Digit Span Forward: adaptivity –;**Attention** by PASAT: adaptivity ↑; **Reasoning** by RAVEN: adaptivity –; **Episodic memory** by RAVLT: adaptivity ↑; **Inhibition** by Stroop: adaptivity –	n.a.	no adjustment	n.a.	n.a.
	**Working Memory Training Task Gain** by maximum gain score as maximum score that individuals reached during Cogmed WM training minus baseline performance: Baseline working memory ↑ (r = 0.62)	n.a.	no adjustment	n.a.	n.a.
Brum et al. (2018)	No significant effects for dose of training reported for any outcome.	n.a.	no adjustment	n.a.	n.a.
	**Verbal Working Memory** by CWMS: ose of training --; by Digit Span Backward: dose of training ↑ (follow-up only); by Letter Number Sequence: dose of training ↓ (both short-term and follow-up); **Visuospatial Working Memory** by Symbol Search: dose of training --; **Verbal Short-term memory** by Digit Span Forward: dose of training --; **Visuospatial Short-term memory** by Spatial Span Forward: dose of training ↓ (follow-up only); by Spatial Span Backward: dose of training ↓ (follow-up only); **Semantic Verbal Fluency:** dose of training ↓ (follow-up only); **Reasoning** by Matrix Reasoning: dose of training ↑ (both short-term and follow-up); **Inhibition** by Stroop Color Interference: dose of training ↑ (both short-term and follow-up)	n.a.	no adjustment	n.a.	n.a.
Heinzel, Lorenz, et al. (2014)	n.a.	**Verbal Working Memory – short-term training gains** by relative n-back training gain as difference of the mean *n*-back performance at posttest and pretest (Δpost-pre) divided by performance at pretest: baseline load-dependent BOLD response pattern of working memory network ↑ (β = 0.598) + gray matter volume ↑ (β = 0.026) + age ↑ (β = 0.095) + sex (β = 0.174) + education ↓ (β = -0.017); baseline load-dependent BOLD response pattern of working memory network ↑ (β = 0.627) + baseline working memory ↑ (β = 0.622) + gray matter volume + age + sex + education (no β reported for covariates)	Varied per model, covariates always included gray matter volume, age, sex, and education	no	yes
	**Verbal Working Memory – short-term training gains** by relative n-back training gain as difference of the mean *n*-back performance at posttest and pretest (Δpost-pre) divided by performance at pretest:	n.a.	no adjustment	n.a.	n.a.

	baseline load-dependent BOLD response pattern of working memory network ↑ (r = 0.589)				
Heinzel, Riemer, et al. (2014)	**Verbal Working Memory Training performance**by n-back task level (Task difficulty was adaptively increased by reducing the interstimulus interval and by increasing the memory load from 2-back up to 5-back): Val/Val ↓	n.a.	Given reports that effects of COMT genotype on working memory performance may be moderated by gender and educational attainment, the authors controlled for these variables in their study. (not further specified)	n.a.	n.a.
Matysiak et al. (2019)	**Verbal Working Memory Training Performance** by maximum n-back-level achieved during each training session: age --; sex --; education --; occupational activity --; baseline working memory ↑ (b = 0.038, SE = 0.183)	n.a.	No, to avoid multicollinearity	n.a.	yes
McAvinue et al. (2013)	**Verbal Short-term memory** by Digit Span Forwards: time spent training ↓ (r = -0.52); **Verbal Short-term memory** by Word Recall Immediate: time spent training -- (r = 0.045 n.s.); **Verbal Long-term memory** by Word Recall Delayed: time spent training -- (r = 0.14 n.s.); **Hospital Anxiety and Depression Scale Post-Training Score:** time spent training ↓ (r = 0.62)	n.a.	no adjustment	n.a.	n.a.
Simon et al. (2018)	n.a.	**Working Memory** by Digit Symbol test: time * adaptivity ↑ * processing speed ?<br>**For all other outcomes** time * adaptivity * age –<br>No further information on the direction and magnitude of the effects was provided by the authors.	adaptivity	no	n.a.
Tusch et al. (2016)	**Verbal Working Memory** by A' discrimination index for n-back stimuli Variable from signal detection theory as speed/accuracy trade-off in processing and diminishing the influence of strategy effects: adaptivity --	n.a.	no adjustment	n.a.	n.a.
	**Verbal Working Memory Training gains:** education –; intelligence --	n.a.	no adjustment	n.a.	n.a.
Weicker et al. (2018)	**Working memory functioning** by composite of span tasks, spatial addition, symbol span, n-back task, PASAT, OSPAN: adaptivity --; **Working memory span** by composite of Span Board and Digit Span: adaptivity ↑ (short-term only); **Visuospatial Working Memory** by Span Board backward: adaptivity ↑ (short-term only); **Executive Functions** composite of Stroop, Trail Making Test and TAP mental flexibility: adaptivity --; **Logical resoning** by Leistungsprüfsystem Subtest 3: adaptivity --; **Long-term verbal memory** by Rex Auditory Verbal Learning test: adaptivity --	n.a.	no adjustment	n.a.	n.a.
	**Working Memory Training gains** by difference of Δpost-pre maximal level of training task: baseline working memory ↑ β = 0.54; baseline verbal working memory ↑ β = 0.63; baseline visuo-spatial working memory ↑ β = 0.68	n.a.	no adjustment	n.a.	n.a.
Zinke et al. (2012)	**Working memory training gains by difference** (Δpost-pre) in each of the trained tasks: baseline working memory ↓	n.a.	no adjustment	n.a.	n.a.

	**Verbal working memory** by digit span backward: baseline working memory ↓ r = –0.59; **Visuospatial Short-term memory** by CORSI block span forward: baseline performance ↓ r = –0.65; by CORSI block span backward: baseline working memory ↓ r = –0.89; by K-ABC: baseline working memory ↓ r = –0.80; **Verbal short-term memory** by digit span forward: baseline performance ↓ r = –0.66	n.a.	no adjustment	n.a.	n.a.
Zinke et al. (2014)	n.a.	Gain scores Δpost-pre<br>**Verbal Working Memory** by subtract-2-span task: age ↓ (β = -0.33)+ intelligence -- (β = 0.06) + baseline working memory ↓ (β = -0.45), by Letter-span plus: 5. age -- (β = -0.16) + intelligence -- (β = -0.06) + vWM gains -- (β = 0.20) + nvWM gains -- (β = 0.22) + ExCon gains ↑ (β = 0.35); **Visuospatial Working Memory** by (modified) K–ABC spatial memory task: age ↓ (β = -0.49) + intelligence -- (β = -0.003) + baseline working memory ↓ (β = -0.59), by CORSI block span: age ↓ (β = -0.35) + intelligence -- (β = 0.16) + vWM gains -- (β = -0.20) + nvWM gains -- (β = 0.25)+ ExCon gains -- (β = -0.03); **Executive Control** by Tower of London task: age -- (β = -0.15) + intelligence ↑ (β = 0.23) + baseline working memory ↓ (β = -0.72), by Tower of Hanoi: age ↓ (β = -0.29) + intelligence -- (β = 0.17) + vWM gains ↓ (β = -0.27) + nvWM gains -- (β = 0.11) + ExCon gains ↑ (β = 0.48); **Fluid Intelligence** by Raven Standard Progressive Matrices: age ↑ (β = 0.44) + intelligence -- (β = -0.17) + vWM gains -- (β = -0.17) + nvWM gains -- (β = 0.13) + ExCon gains -- (β = 0.17); **Inhibition** by Stroop interference: age ↓ (β = -0.36) + intelligence ↑ (β = 0.26) + vWM gains -- (β = -0.16) + nvWM gains -- (β = -0.04) + ExCon gains -- (β = -0.13)	Varied per model. Age and intelligence always included.	no	n.a.

Note. Within prediction results, ↑ indicates positive predictors, i.e. higher values in the predictor variable are associated with better training outcomes, ↓ indicates negative predictors, i.e. lower values in the predictor variable are associated with better training outcomes, -- indicates non-significant relationships between predictor and training outcome, and ? indicates that a predictor was investigated, but results were not reported appropriately. For prognostic model studies, only the final models reported in the original manuscript are reported.
